# GDBr: genomic signature interpretation tool for DNA double-strand break repair mechanisms

**DOI:** 10.1093/nar/gkae1295

**Published:** 2025-01-11

**Authors:** Hyunwoo Ryu, Hyunho Han, Chuna Kim, Jun Kim

**Affiliations:** Division of Biotechnology, College of Life Sciences and Biotechnology, Korea University, 145, Anam-ro, Seongbuk-gu, Seoul 02841, Republic of Korea; Department of Computer Science and Engineering, Korea University, 145, Anam-ro, Seongbuk-gu, Seoul 02841, Republic of Korea; Department of Urology, Urological Science Institute, Yonsei University College of Medicine, 565, Seongsan-ro, Seodaemun-gu, Seoul 03721, Republic of Korea; Aging Convergence Research Center, Korea Research Institute of Bioscience and Biotechnology, 125, Gwahak-ro, Yuseong-gu, Daejeon 34141, Republic of Korea; Department of Bioinformatics, KRIBB School of Bioscience, Korea University of Science and Technology (UST), 217, Gajeong-ro, Yuseong-gu, Daejeon 34113, Republic of Korea; Department of Convergent Bioscience and Informatics, College of Bioscience and Biotechnology, Chungnam National University, 99, Daehak-ro, Yuseong-gu, Daejeon 34134, Republic of Korea; Graduate School of Life Sciences, College of Bioscience and Biotechnology, Chungnam National University, 99, Daehak-ro, Yuseong-gu, Daejeon 34134, Republic of Korea

## Abstract

Large genetic variants can be generated via homologous recombination (HR), such as polymerase theta-mediated end joining (TMEJ) or single-strand annealing (SSA). Given that these HR-based mechanisms leave specific genomic signatures, we developed GDBr, a genomic signature interpretation tool for DNA double-strand break repair mechanisms using high-quality genome assemblies. We applied GDBr to a draft human pangenome reference. We found that 78.1% of non-repetitive insertions and deletions and 11.0% of non-repetitive complex substitutions contained specific signatures. Of these, we interpreted that 98.7% and 1.3% of the insertions and deletions were generated via TMEJ and SSA, respectively, and all complex substitutions via TMEJ. Since population-level pangenome datasets are being dramatically accumulated, GDBr can provide mechanistic insights into how variants are formed. GDBr is available on GitHub at https://github.com/Chemical118/GDBr.

## Graphical Abstract

**Graphical Abstract:**
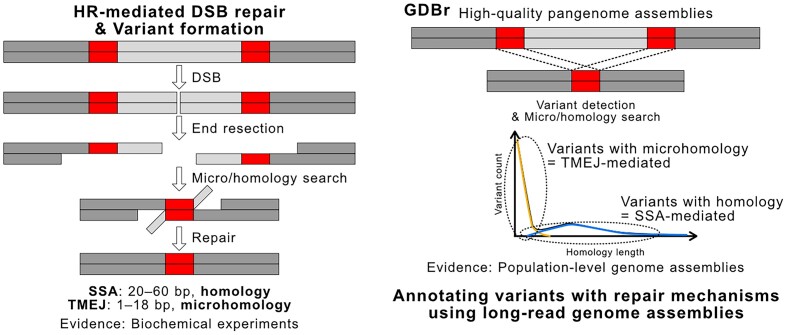

## Introduction

Stable maintenance and transmission of the genome is critical for the integrity of species; nonetheless, DNA is continuously damaged, sometimes leading to double-strand breaks (DSBs) (1). Several mechanisms have evolved to handle and repair these DSBs (2–5), but the repair process is typically not perfect, changing the original DNA sequences and causing mutations in the genome (1,6). These mutations, or genetic variants, can increase genetic diversity to better adapt to novel environments and occur in cancer cells (7–12). Thus, it is important to understand how specific DSB repair mechanisms produce these mutations. However, the lack of both high-quality genomic resources and computational tools to interpret repair processes based on mutational signatures limits this endeavour.

**Figure 1.**
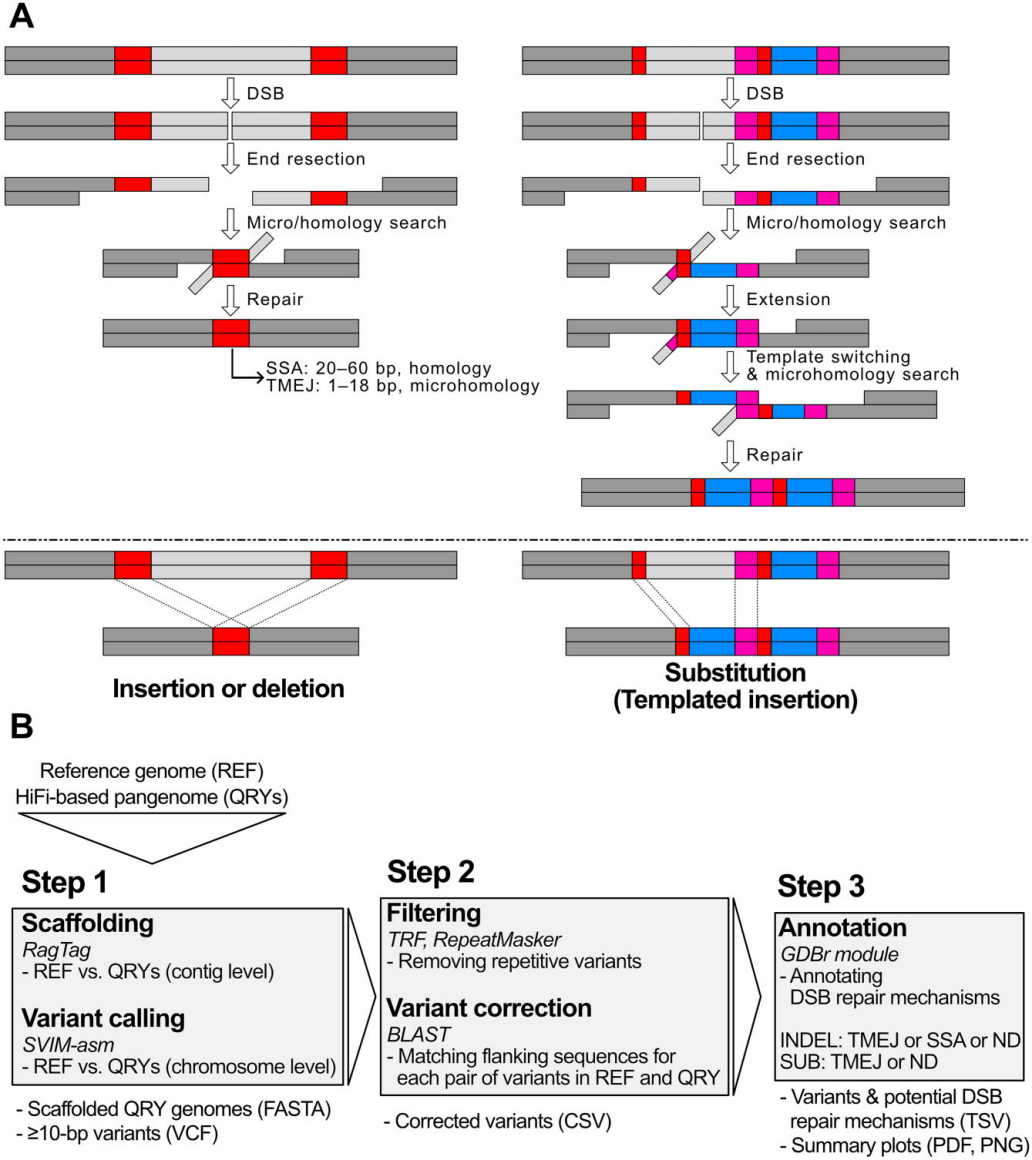
Genomic signatures generated by HR-mediated DSB repair mechanisms and the GDBr pipeline. (**A**) Schematic representation of HR-mediated genomic signatures. Left: How TMEJ or SSA forms an indel. DNA is damaged by DSB, and then repair starts with end resection of both flanking sequences. Next, exposed single-stranded ends are compared to identify micro/homology (red box), and both flanking sequences are annealed and completely repaired. TMEJ and SSA differ in their micro/homology length. The resulting indel contains micro/homology near its breakpoint. Right: How TMEJ creates a complex substitution, also known as templated insertion. DSB, end resection, microhomology search and DNA synthesis occur first, but the repair process is not completed. The synthesized and extended end sometimes can switch its template to search another microhomology, before finishing the repair. After TMEJ-mediated repair, a complex substitution can be created, so that the breakpoint contains two microhomologous sequences and a homologous inserted sequence. Red and magenta boxes represent the first (smaller red box) and second (larger magenta box) microhomology sequences, respectively, and the blue box represents the sequence generated by templated insertion. (**B**) Overview of the GDBr pipeline. GDBr uses a reference genome and HiFi-based query genome assemblies as input files. First, query genome assemblies are compared with the reference genome and scaffolded to identify the corresponding chromosomes using RagTag. Genetic variants in each chromosome were called using SVIM-asm. Second, repetitive variants were removed using TRF and RepeatMasker. Non-repetitive variants were corrected and searched using BLAST to identify the corresponding positions of each variant in the reference and query genomes. Third, a specific DSB repair mechanism of each variant is annotated using its genomic signature. GDBr outputs tables for variants as well as their corresponding DSB repair mechanisms and summary plots for genomic signatures.

Major DSB repair mechanisms are well conserved from yeasts to humans and leave distinguishable mutational footprints in the genome. For example, non-homologous end joining (NHEJ) causes small random insertions or deletions (indels; ∼1–5 bp) after DSB repair (4,13,14). In contrast, several homologous recombination (HR) mechanisms can leave genetic variants of ≥10 bp because they use extensive single-strand end resection during DSB repair (15,16) (Figure 1A). Polymerase theta-mediated end joining (TMEJ) and single-strand annealing (SSA) are HR-mediated mechanisms. Their exposed single-stranded ends are annealed to repair the DSB by either microhomology or homology (micro/homology) searches (17,18) (Figure 1A). The genetic variants generated by TMEJ or SSA can be distinguished because TMEJ requires microhomology (2–18 bp), whereas SSA requires longer homology (20–60 bp) (2,19–22). Thus, these sequence-level genomic signatures of DSB repair mechanisms allow retrospective identification of the underlying DSB repair mechanisms of genetic variants from genomic data. However, the number of variants formed via TMEJ or SSA in the population remains unknown.

Technical limitations have hindered inferring the DSB repair mechanisms of genetic variants. Although short-read sequencing technologies allow identifying single-nucleotide polymorphisms, these technologies are not suitable for detecting large variants and their exact breakpoints (23–26). Long-read sequencing technologies have resolved these limitations by providing long (>10 kb) and accurate (∼99.9%) DNA information at an affordable price (∼1000 USD for library preparation and sequencing run) (27–29). In addition, these long reads can be further assembled into larger (∼10 Mb of N50) and more accurate (QV 50–60) contigs (30–34), which allows to precisely detect genetic variants with exact breakpoint sequences at pangenome level. Using these technical advances, we could reliably infer the underlying DSB repair mechanisms of genetic variants through their genomic signatures.

Here, we report a novel computational tool, GDBr, that helps infer DSB repair mechanisms of non-repetitive genetic variants using micro/homology. GDBr works via (i) variant calling, (ii) variant correction and filtering, and (iii) DSB repair mechanism annotation followed by visualization. To benchmark GDBr performance, we used a draft human pangenome reference dataset that includes 94 phased genome assemblies of 47 individuals (35). GDBr provides an opportunity to understand the mechanisms generating genetic variants in any population.

## Materials and methods

### Background information and algorithmic schemes for detection of genomic signatures from HR-based DSB repair mechanisms

We used the micro/homology in flanking sequences of variants to identify HR-mediated DSB repair signatures (Figure 1A). The detailed workflow of GDBr is illustrated in Supplementary Figure S1. For indels generated by SSA and TMEJ, both flanking sequences in each variant should overlap in the original genome as they were used for micro/homology searching and annealing (Figure 1A, left). For complex substitutions (Sequence Ontology, SO:1 000 005) (36) generated by TMEJ (i.e. templated insertions), the left and right flanking sequences originate from different micro/homology searching events, so these flanking and substituted sequences should be identified as a chunk near the breakpoints of the original genome (Figure 1A, right; additional examples of templated insertions are illustrated in Supplementary Figure S2). SSA and TMEJ could be further separated based on the length of flanking sequences. GDBr uses these characteristics to infer the DSB repair mechanisms of non-repetitive variants. To annotate DSB repair mechanisms, GDBr works through three major steps (Figure 1B).

It is important to note that the input for GDBr is assembled genomes, not raw sequencing reads. Additionally, it is strongly recommended to use genomes assembled with high-fidelity long-read sequencing data. This is because GDBr compares the two flanking sequences of each variant to determine whether they exhibit micro/homology genomic signatures. Using highly accurate genome assemblies ensures the reliability of this signature detection, whereas inaccurate genomes may lead to misannotation of repair mechanisms.

*Step 1: Pre-processing for variant calling*. First, GDBr uses a chromosome-level reference genome and query genome(s) as input files and scaffolds contigs into chromosomes using RagTag (version v2.1.0; *ragtag.py scaffold -u -C*) (37). This step assigns chromosome names to sets of contigs and allows chromosome-to-chromosome comparison. Subsequently, GDBr calls ≥10-bp indels as a raw variant set using SVIM-asm (version 1.0.3; *svim-asm haploid --min_sv_size 10 --tandem_duplications_as_insertions --interspersed_duplications_as_insertions*) (38). Note that duplication variants are called insertions in GDBr and memory usage can be adjusted using the *--low-memory* option in this GDBr step.

*Step 2: Variant correction and filtering*. These raw indels do not have enough information to infer DSB repair mechanisms because SVIM-asm does not output complex substitutions and variant positions in the query genome. In addition, repetitive variants are not suitable for inferring DSB repair mechanisms, as the same repetitive variant can be formed through different mechanisms. To resolve these limitations and potential errors in the SVIM-asm, raw indels were processed to identify and determine the exact positions of variants in the reference and query genomes and to remove repetitive variants as follows. First, we analysed whether raw indels or their 50-bp flanking sequences contained repetitive sequences or not using TRF (version 4.09.1; *trf 2 5 7 80 10 50 500*) and RepeatMasker (version 4.1.5; *default option*) to remove repetitive sequences (39,40). Since TRF works much faster than RepeatMasker, TRF was used first and RepeatMasker was then applied to identify any species-specific repetitive sequences, including transposable elements. The default length for repetitive sequence search in GDBr is set to 50 bp, but can be adjusted using the parameter *--repeat_find_len*. We then extracted 2-kb flanking sequences from both ends of each variant in the reference genome using pyfaidx (version 0.7.2.1) (41). To identify their query positions, these flanking sequences were searched in the query genome using BLASTn (version 2.14.0; *blastn -task megablast -strand plus*) (42,43).

After this search, variants were further corrected and classified as insertions, deletions, complex substitutions and exceptions using the following logic (Supplementary Figure S1). For deletions, flanking sequences should be continuously located in the query genome. For insertions, flanking sequences should be separated and their distance should be the same as the length of variants in the query genome. In that case, these variants were considered to be called correctly and classified as corrected deletions and insertions, respectively; next, their positions in the reference and query genomes were saved.

If not the case, flanking sequences for deletions and insertions should overlap or be separated in the query genome because of variant miscalling by SVIM-asm. As these variants might have been miscalled in terms of their type and/or position, we corrected them as follows. For miscalled variants whose flanking sequences overlapped in the query genome, we trimmed off the overlapping sequences from the original 2-kb flanking sequences for the initial search and repeated the search for these trimmed flanking sequences to acquire the correct variant positions. We then repeated this step three times to test whether flanking sequences overlap. When no overlap existed, these variants and their corrected positions were saved. However, if these flanking sequences still overlapped, we considered these variants difficult to correct and categorized them as exceptions.

After this initial correction, we obtained all initially corrected variants and processed them to finalize variant information as follows: (i) If the final flanking sequences were separated in the reference genome but were continuously located in the query genome, we categorized these variants as corrected deletions. (ii) If the final flanking sequences were continuously located in the reference genome but were separated in the query genome, we categorized these variants as corrected insertions. (iii) If the final flanking sequences were separated in both the reference and query genomes, we categorized these variants as complex substitution candidates. Note that some complex substitution candidates were actually not true complex substitutions, but indels, as one substituted sequence contains the other substituted sequence (e.g. ATGCT…GC to AT). Thus, we verified complex substitution candidates using BLASTn and categorized such cases as indels. Otherwise, the remaining complex substitution candidates were classified as corrected complex substitutions (Supplementary Figure S1).

These flanking sequences were used for specifically locating variant positions in the reference and query genomes. While longer flanking sequences can provide more specificity, they also demand greater computational resources, leading to slower processing. Additionally, excessively long flanking sequences may contain repetitive sequences, leading to misidentification of variants as repetitive. To optimize this trade-off, we tested flanking sequence lengths of 500, 1000, 2000, 4000 and 8000 bp. We found that 2000 bp was the optimal choice, offering similar specificity to 4000 bp while avoiding excessive computational demands (Supplementary Table S1). In addition, these flanking sequences may overlap if the variant is a complex substitution or repetitive. We repeated this search three times to determine which case applies. If flanking sequences continue to overlap after three rounds of searching, GDBr classifies the variant as repetitive rather than a non-repetitive indel or complex substitution. It is noteworthy that the length of flanking sequences can be adjusted using the parameter *--sv_find_len* in GDBr (Supplementary Table S2).

The corrected indels and complex substitutions and their 50-bp flanking sequences were reanalysed using TRF (version 4.09.1; *trf 2 5 7 80 10 50 500*) to completely remove repetitive sequences (39). The output, containing variant type information such as insertion, deletion, complex substitution, repeat and exception, was then saved in CSV (comma-separated values) format.

*Step 3: Annotation of DSB repair mechanisms*. Corrected and filtered variants (insertions, deletions and complex substitutions) were annotated to identify potential underlying DSB repair mechanisms as follows. For indels, we assessed whether the flanking sequences of each variant exhibited micro/homology signatures using BLASTn (version 2.14.0; *blastn -task megablast -strand plus*) (42,43). Note that BLASTn allows mismatches in micro/homology. GDBr does not specify which sequences are ancestral or derived for each indel. However, in theory, for deletions with micro/homology, the reference sequences are considered ancestral, and the query sequences are derived; the reverse is true for insertions. If a variant exhibited micro/homology, we annotated TMEJ or SSA based on micro/homology length distribution. For complex substitutions, we similarly analysed whether the flanking sequence exhibits micro/homology patterns and whether their substituted sequences can be identified near the original loci. If both conditions were true, we annotated them as TMEJ-mediated templated insertions. We removed tandem duplications by analysing whether homology lengths were >90% of variant sizes. This step produces a TSV (tab-separated values) file containing the reference position, query position, variant type, annotated DSB repair mechanism, homology length and homology sequence of each variant. The output can also be supported by a BED (browser extensible data) file that contains only reference positions and homology lengths.

For steps 1 and 2, raw variants, corrected variants and filtered variants were automatically counted and visualized as stacked bar graphs. If existing, their micro/homology length distributions were automatically drawn as histograms. Since TMEJ and SSA have substantially distinct micro/homology length distributions, the merged micro/homology length distribution of variants was separated based on a local minimum point identified by local regression, resulting in distinguishable TMEJ and SSA distributions. The two distributions were further modelled using Poisson distributions, implemented with the *scipy.optimize* function from the SciPy Python package (version 1.10.0), with the assumption that TMEJ and SSA each follow independent Poisson distributions. Their respective lambda parameters were constrained within ranges of 5–20 for TMEJ and 37–50 for SSA. Moreover, the density of these annotated variants along chromosomes was visualized in a histogram.

### Application of GDBr to the draft human pangenome

To understand the underlying DSB repair mechanisms of human genetic variants, we applied GDBr using T2T-CHM13 as a reference genome and draft human pangenome assemblies as query genomes (35,44). The draft human pangenome contains 94 human genome assemblies from 47 diploid human individuals. For GDBr, the T2T-CHM13 genome serves as a superior reference compared to the GRCh38 genome. This is primarily because the T2T-CHM13 genome was constructed using high-fidelity long-read sequencing data, whereas the GRCh38 genome was not. Advances in high-fidelity long-read sequencing have enabled the T2T-CHM13 genome and draft human pangenome assemblies to more effectively resolve complex genomic regions, resulting in a greater number of sequences compared to the GRCh38 genome. If the GRCh38 genome were used as the reference, these well-resolved and accurately assembled sequences might be misidentified as insertions or other variants. To prevent this issue, we chose the T2T-CHM13 genome as the reference for GDBr. We collapsed these variants to count singletons and shared variants among assemblies using SURVIVOR (version 1.0.6; *SURVIVOR merge sample_files_list 100 1 1 1 0 1 sample_merged.vcf*) (45).

For our GDBr benchmarking analysis, we used a modern computer with an OS of Ubuntu 20.04.6 LTS, dual-Intel^®^ Xeon^®^ Platinum 8360Y @ 2.4 GHz (72 cores, 144 threads), 512 GB RAM and RAID6 HDD. SSD is not necessary to run GDBr, as it only improves the processing speed by ∼3%. CPU usage and time were analysed using GNU time (version 1.8; default option) and memory usage was analysed using psutil (version 5.9.0). This benchmarking experiment can be replicated in GDBr using the *--benchmark* option.

## Results

### GDBr benchmark

GDBr was designed to annotate the DSB repair mechanisms of pangenome level variants based on efficient parallelization. In our benchmarking analysis using a modern computer with 144 threads and 512 GB RAM, each human genome was processed in ∼10 min and the 94 human genome assemblies were processed in ∼16 h with maximum computational resources (Table 1). With the low memory usage mode of GDBr, ∼40 min and 65 h are needed for 1 and 94 human genome assemblies, respectively (Table 1). GDBr also exhibited high multiprocessing efficiency, 85% for the correct step (the bottleneck step) and >50% for the other steps with maximum computational resources, for instance (Table 1). The finalized output is shown in Supplementary Table S1.

**Table 1.** GDBr benchmark results for processing the human draft pangenome and a single human genome

Data type	Step	Wall clock time (h:mm:ss)	CPU time (h:mm:ss)	Multiprocessing efficiency (%)	Allocated number of threads	Peak memory (GB)
Human pangenome	Pre-process (*default*)	04:19:09	173:03:32	55.6	72	462.0
	Pre-process *(--low_memory*)	54:51:51	180:45:53	4.6	72	34.7
	Correct	11:11:07	1351:03:39	85.1	144	54.1
	Annotate	00:06:23	07:40:40	50.8	144	27.0
Single human	Pre-process	00:39:52	02:02:45	4.3	72	31.7
genome	Correct	00:06:32	12:14:10	77.9	144	44.6
(HG002.1)	Annotate	00:01:06	00:09:50	6.1	144	23.5

### Pre-processing of the draft human pangenome using GDBr

**Figure 2.**
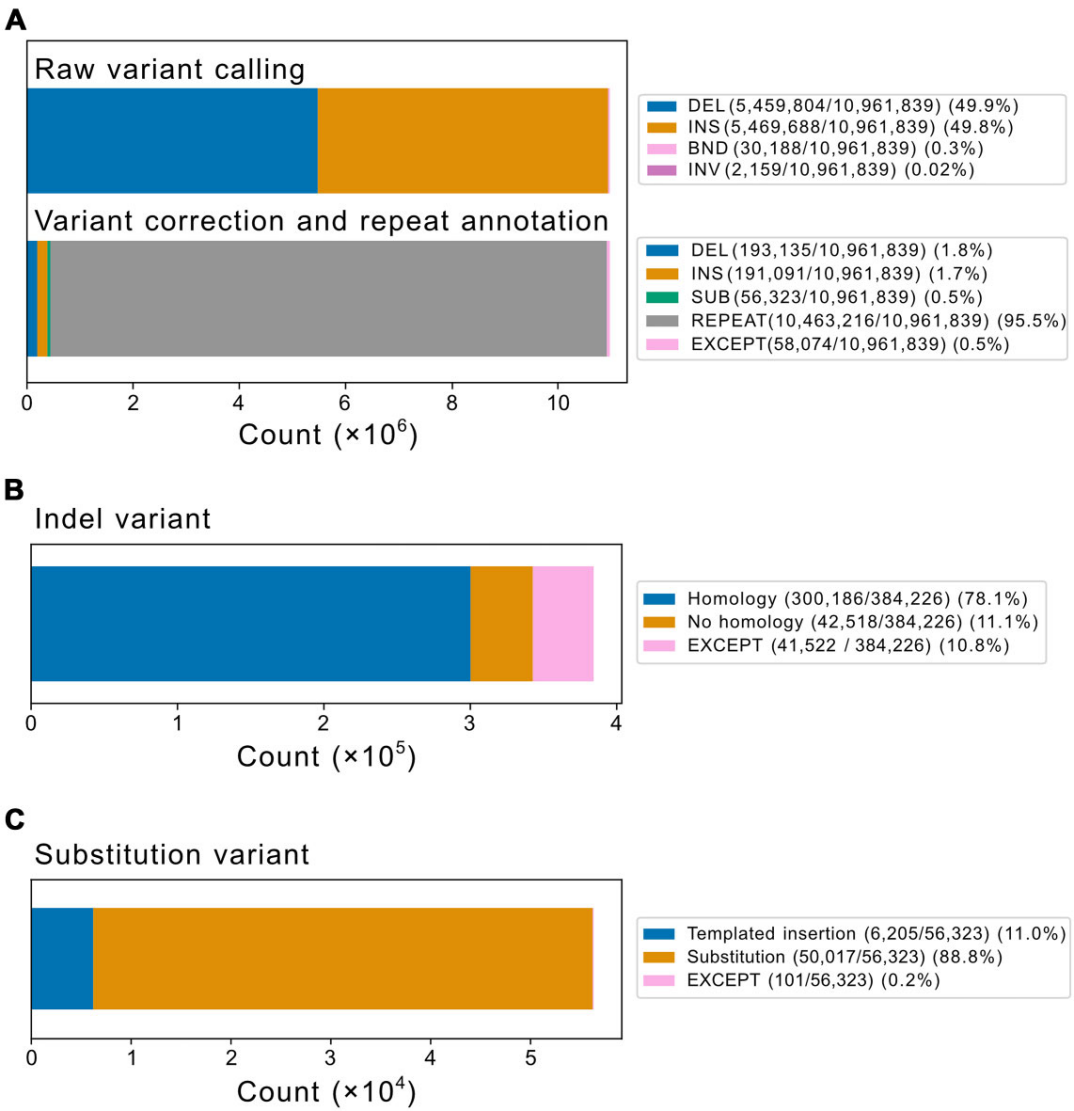
Human pangenome variant classification using GDBr. (**A**) Stacked bar graphs for raw variants (upper panel) and pre-processed variants (lower panel). Presence/absence patterns of micro/homology analysed for corrected, non-repetitive indels (**B**) and complex substitutions (**C**).

Using GDBr, we first called ≥10-bp raw variants in 94 human genome assemblies, obtaining 10 961 839 variants, including 5 469 688 insertions and 5 459 804 deletions (99.7%) (Figure 2A). These variants were then pre-processed to correct miscalling and remove repetitive sequences. Of the 10 961 839 variants, 95.5% (10 463 216 variants) contained repetitive sequence; the remaining 466 278 non-repetitive variants were categorized as 193 135 (41.4%) insertions, 191 091 (41.0%) deletions and 56 323 (12.1%) complex substitutions (Figure 2A and B; see Supplementary Table S1 for the corresponding data of each genome assembly). Next, these non-repetitive insertions, deletions and complex substitutions were analysed to infer their underlying DSB repair mechanisms.

To annotate DSB repair mechanisms, we analysed micro/homology genomic signatures for indels and templated insertion signatures for complex substitutions. Out of the 384 226 indels, 42 518 variants were categorized as tandem duplications and were therefore removed. The final total of 342 704 indels and 56 222 complex substitutions represents the sum of variants found across all genome assemblies. Each individual genome assembly contains ∼116 271 indels and 321 complex substitutions, which may collapse to around 27 723 indels and 207 complex substitutions, as some variants are shared among the different genome assemblies (Supplementary Figure S2). Of these variants, 23.8% and 64.6% of indels and complex substitutions were singletons, respectively.

Of the remaining 342 704 indels, 300 186 (87.6%) exhibited micro/homology signatures and only 42 518 (12.4%) did not (Figure 2C). In addition, all 94 genome assemblies exhibited similar ratios of micro/homology (86.7–88.7% for micro/homology indels and 8.8–13.6% for non-micro/homology indels; Supplementary Figure S3 and Supplementary Table S1). This indicates that TMEJ- or SSA-mediated DSB repair events frequently occurred in the human genome to form most non-repetitive indels. Among the 56 323 complex substitutions, only 101 complex substitutions (0.2%) were tandem duplications, and of the remaining 56 222 complex substitutions, 6205 (11.0%) complex substitutions exhibited templated insertion signatures with micro/homology patterns and 50 017 (89.0%) did not (Figure 2C, Supplementary Figure S3 and Supplementary Table S1). This implies that TMEJ-mediated templated insertion is minor and the possible existence of other major complex substitution-forming mechanism(s).

### Inferring DSB repair mechanisms using micro/homology lengths

**Figure 3.**
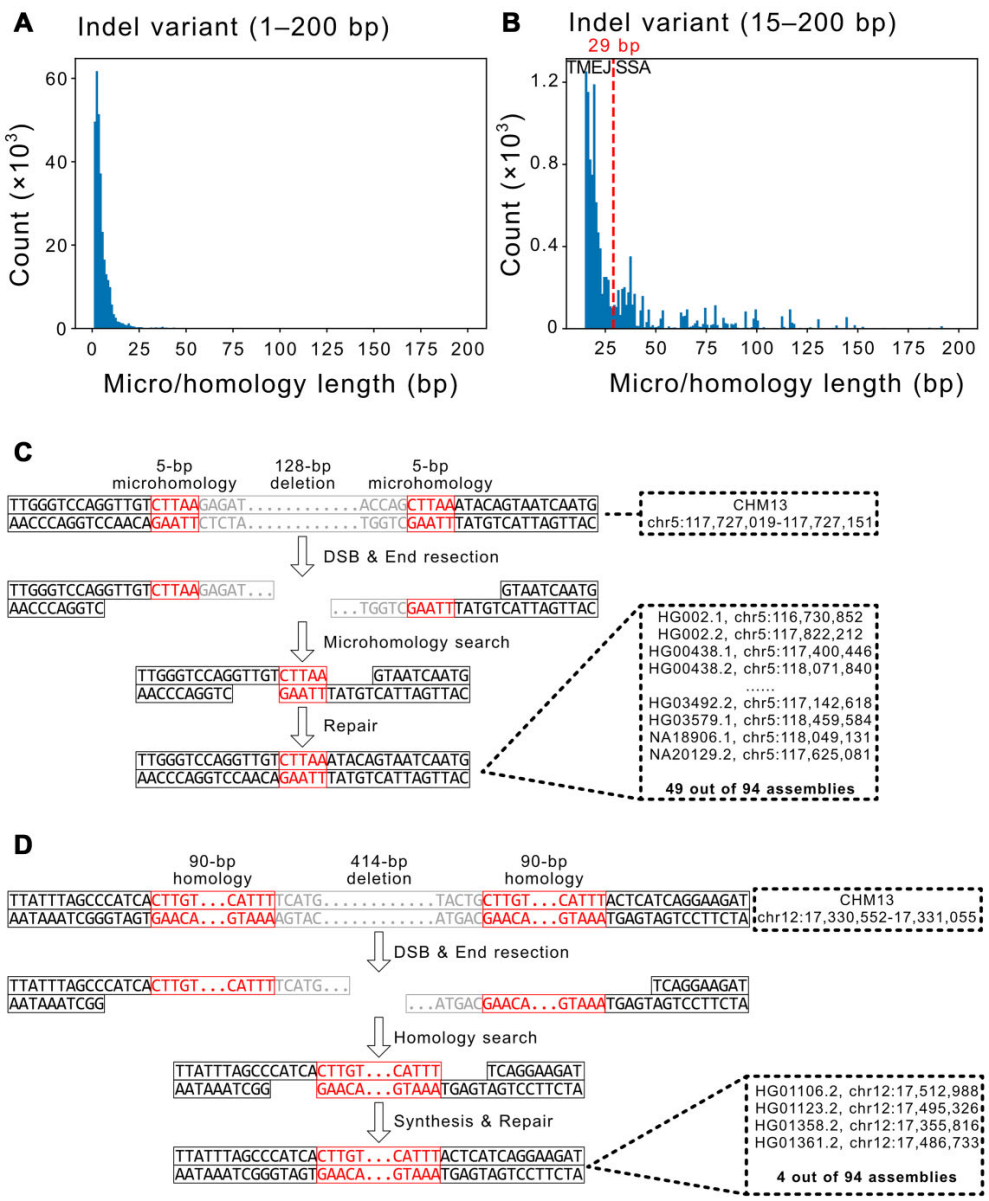
Micro/homology length distribution of indels and representative examination of potential DSB repair mechanisms. Micro/homology length distribution of indels in the 1–200-bp (**A**) and 15–200-bp (**B**) ranges. The red vertical dotted line indicates a putative border line for dividing variants generated via TMEJ (left) and SSA (right). Representative images of (**C**) TMEJ- and (**D**) SSA-mediated indels detected in the draft human pangenome data. Black boxes represent sequences near the variant not changed during the repair process. Red boxes represent micro/homology sequences. Grey boxes represent deleted sequences during end resection. Dotted boxes show variant positions in the reference T2T-CHM13 and query genomes.

**Figure 4.**
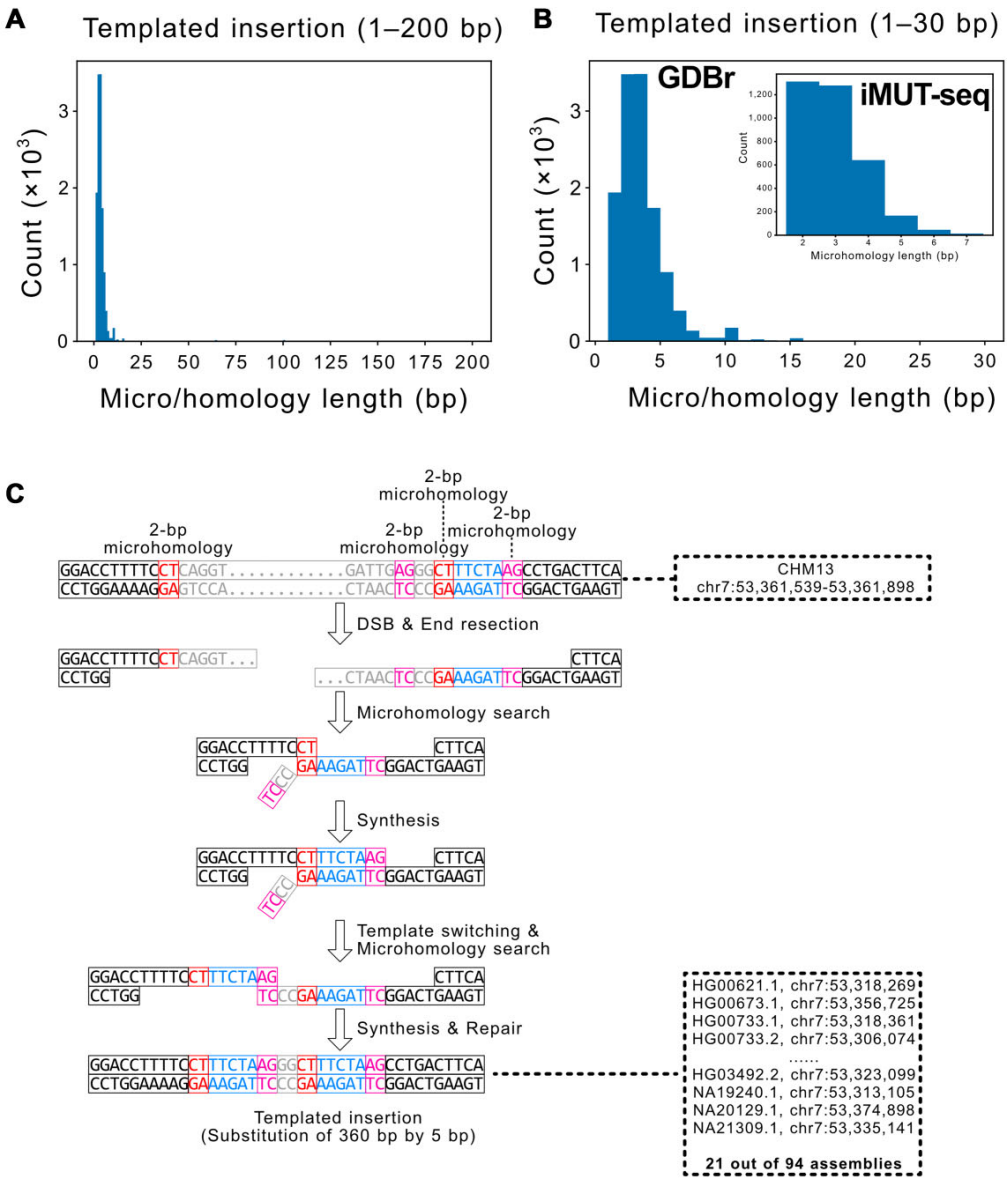
Micro/homology length distribution of complex substitutions and a representative example. Microhomology length distribution of complex substitutions representing the 1–200 bp (**A**) and 1–30 bp (**B**) ranges. In panel (B), GDBr represents the distribution from this study, while iMUT-seq represents the distribution obtained from biochemical experiments (24). (**C**) Representative image of a complex substitution or templated insertion variant. Black boxes represent sequences near the variant not changed during repair. Red and magenta boxes indicate the first and second microhomology sequences, respectively. Grey boxes represent deleted sequences during end resection. Dotted boxes show variant positions in the reference T2T-CHM13 and query genomes.

To precisely annotate their underlying mechanisms, we visualized the micro/homology length distribution of non-repetitive, micro/homology-mediated variants. Impressively, most indels exhibited <15-bp microhomology (288 455 out of the 300 185 indels; 96.1%) as did almost all complex substitutions (6165 out of the 6205 complex substitutions; 99.2%; Figures 3 and 4; Figures 3C and 4C present representative examples of TMEJ- and SSA-mediated variants; additional examples of indels and complex substitutions generated by TMEJ and SSA are illustrated in Supplementary Figure S4). The peaks of both indels and complex substitutions were within the 2-bp microhomology length. Indels also exhibited micro/homology >15 bp, but complex substitutions did not. It is important to note that the microhomology length distributions identified by GDBr were consistent with experimentally measured distributions from DSB-induced cells repaired via TMEJ (Figure 4B) (24). This evidence supports that most indels and complex substitutions with micro/homology would result from TMEJ-mediated DSB repair events, rather than SSA, which requires much longer homology.

Next, we attempted to separate TMEJ- or SSA-mediated repair events based solely on the micro/homology length distribution. Note that TMEJ typically requires 2–18-bp microhomology and SSA requires 20–60-bp longer homology (2,19–22) and our results revealed that the first peak of the micro/homology length distribution was 2 bp (Figure 3A). Thus, we hypothesized that the 2-bp peak is generated by TMEJ-mediated DSB repair events and attempted to identify the peak for SSA-mediated DSB repair events by more precisely focusing on the 15–200-bp region. Interestingly, we identified a peak near 35 bp, and the micro/homology distribution can be separated at 29 bp (Figure 3B): ≤29 bp, TMEJ would be the responsible DSB repair mechanism; and >29 bp, it would be SSA, as indicated by previous biochemical experiments (2,46,47). Accordingly, we inferred that 296 323 indels (98.7%, ≤29 bp) would result from TMEJ and 3863 indels (1.3%, >29 bp) from SSA (98.3–99.1% for TMEJ and 0.9–1.7% for SSA in each genome assembly; Figure 3B, Supplementary Figure S3 and Supplementary Table S1). Since the two distributions slightly overlap, GDBr may misannotate the underlying repair mechanisms near the 29-bp baseline. To estimate the number of variants potentially misannotated, we calculated the overlap between these distributions (Supplementary Figure S5). Our analysis revealed that only 0.003% of the TMEJ-mediated distribution and 5.3% of the SSA-mediated distribution exceeded the baseline.

We then analysed how this result could be influenced by BLASTn parameters. First, we tested four BLASTn search modes: *blastn-short*, *megablast*, *blastn* and *dc-megablast*. Overall, these search modes yielded very similar results, with *megablast* showing intermediate results between *blastn-short* and *blastn*, as well as *dc-megablast* (Supplementary Figure S6). Notably, *blastn* and *dc-megablast* produced identical results, likely due to their nearly identical parameter combinations for BLASTn (42,48). For TMEJ, the microhomology length identification using *megablast* was consistent with that of *blastn-short* and *blastn* at 98.7% and 97.6%, respectively (Supplementary Figure S6). For SSA, homology length identification ratios aligned with *blastn-short* and *blastn* at 90.3% and 91.6%, respectively (Supplementary Figure S6). The main differences between *blastn-short* and *blastn*, compared to megablast, involved the classification of TMEJ as ‘Not Determined’ (2.3% and 1.7%, respectively) and the identification of SSA as TMEJ in *blastn-short* (9.2%) or as ‘Not Determined’ in *blastn* (13.7%) (Supplementary Figure S6). These opposite discrepancies in SSA identification likely stem from the fact that *blastn-short* is designed for short DNA sequence comparisons, which can result in the misannotation of homology as slightly shorter microhomology. In contrast, *blastn* imposes overall rewards that are too low for homologous sequences, as it was designed for interspecies comparisons, leading to the loss of true homology sequences. Based on these observations, we recommend using *megablast* as the default option for GDBr.

We also tested the effect of sequence length on repetitive sequence search by repeating the procedure with values of 0, 10, 20, 30, 40 and 50 for *--repeat_find_len* in GDBr (Supplementary Figure S7). With a setting of 0, non-repetitive and repetitive variants constituted 40.4% and 55.7% of the total variants, respectively (Supplementary Figure S7). However, 65.8% of these non-repetitive variants consisted of tandem duplications rather than variants with micro/homology (Supplementary Figure S7), suggesting potential false positives for micro/homology detection. Notably, this setting achieved a micro/homology separation baseline of 31 bp, aligning more closely with known values. At 10 bp, the proportion of repetitive variants increased significantly, with non-repetitive and repetitive variants at 12.3% and 85.3%, respectively. Its tandem duplication ratio was still 42.6% (Supplementary Figure S7). The tandem duplication ratios for 20, 30, 40 and 50 were 24.1%, 16.1%, 12.0% and 11.1%, respectively (Supplementary Figure S7). For this study, we selected 50 bp to minimize potential false positives, as this setting showed a significantly reduced occurrence of tandem duplications and a convergence of the separation baseline near 30 bp. However, users can adjust this parameter based on their experimental objectives. Even at lower values such as 10 and 20 bp, GDBr can analyse draft pangenome data within 55 and 23 h, respectively (Supplementary Table S3).

To test whether chromosomal position influences the type of DSB repair mechanism involved, we analysed the chromosomal distributions of TMEJ-mediated indels, SSA-mediated indels and non-micro/homology indels. We showed that these indels exhibited similar chromosomal distributions, suggesting that chromosomal locations do not affect the mechanism involved (Supplementary Figure S8).

## Discussion

The process of DSB repair is sometimes mutagenic, leading to variant formation. Currently, a huge amount of whole-genome sequencing data has been accumulated to identify many genetic variants and several methods have been developed for understanding of their specific repair mechanisms (24,49,50). In particular, short-read sequencing has been widely used for this purpose, but its short-read lengths, typically ∼150 bp, interrupt the identification of large genetic variants and long homology sequences near them (51–54). Error-prone long-read sequencing has been used to solve this problem but focusing on a few variants by manual investigation or a single repair mechanism, such as TMEJ (8,10,55). Moreover, previously established biochemical evidence supports that TMEJ and SSA can be achieved by 2–18-bp or 20–60-bp micro/homology sequences, but this has not been thoroughly tested in genetic variants in the human population (2). Using a draft human pangenome reference and GDBr, we uncovered the possible DSB repair mechanisms underlying human genetic variants.

The unique contribution of GDBr lies in its ability to utilize high-quality genome assemblies generated from long-read sequencing technologies to annotate potential repair mechanisms for non-repetitive long variants. While previous approaches have relied on short-read sequencing or earlier long-read technologies, these methods lacked the precision necessary for accurately identifying long variants and their flanking sequences.

GDBr leverages the advancements in long-read sequencing, such as those provided by the Human Pangenome Reference Consortium, to comprehensively detect and analyse longer variants in the genome with higher accuracy. This advancement allows GDBr to infer repair mechanisms, particularly HR signatures, which were previously limited or prone to error due to lower-quality data. To the best of our knowledge, GDBr is the first tool to annotate these repair mechanisms, corresponding to known biochemical processes, based on high-fidelity genome assemblies.

GDBr showed that most non-repetitive indels and complex substitutions in the human population were probably generated via TMEJ-mediated repair events, as 98.7% of indels and 100% complex substitutions exhibited microhomology genomic signatures. This high proportion of TMEJ in non-repetitive indels aligns with existing knowledge, suggesting that TMEJ, rather than SSA, serves as the primary mechanism for repairing DSBs with resected ends in eukaryotes (10,21,50,56,57). In addition, since templated insertions are solely formed via TMEJ, they are indicative of it (5). Our templated insertion-type complex substitutions did not exhibit long homology but short microhomology genomic signatures. Moreover, the microhomology length distributions identified by GDBr in the draft human pangenome are very similar to the experimentally measured microhomology length distribution of TMEJ-mediated repair events after DSB induction (Figure 4B) (24). The agreement with previous biochemical experiments demonstrates that GDBr can effectively annotate DSB repair mechanisms. It should be noted that TMEJ- and SSA-mediated distributions could overlap, but this overlapping region would be negligible. Thus, GDBr annotations will be reliable for the majority of the repair mechanisms underlying the variants.

It appears that TMEJ and SSA favour distinct yet specific lengths of micro/homology for effective repair, as indicated by their distribution peaks. These peaks are located around 2 bp for TMEJ and ∼35 bp for SSA, which aligns with prior biochemical knowledge (2,19–22). Additionally, TMEJ may utilize microhomology sequences of 2–30 bp, while SSA may rely on homology sequences of 30–150 bp. These distributions are slightly different, but largely consistent with the known ranges. Our microhomology distribution overlaps primarily with the known TMEJ-mediated distribution (2–18 bp), while our homology distribution aligns with the known SSA-mediated distribution (20–60 bp). These two distributions show slight overlap, as the microhomology-mediated distribution has a long tail. This long tail may explain our higher baseline (∼29 bp) compared to previous estimates (∼20 bp), but overall, these distributions remain consistent with prior knowledge.

It remains unclear whether these distributions are due to the enzymatic efficiency of TMEJ and SSA, the prevalence of micro/homology sequences in the human genome or a combination of both factors. Given that the frequency of micro/homology in flanking sequences decreases less sharply than expected, these distributions may not be solely explained by sequence occurrence in the human genome. A single-base increase in micro/homology length could theoretically reduce its frequency in the genome by up to 4-fold. These hypotheses should be further validated through biochemical experiments.

Although GDBr could annotate HR-mediated DSB repair mechanisms of genetic variants, it could not uncover all variants in the genome and identify the underlying repair mechanisms. NHEJ is probably the major repair mechanism of DSBs, but it is difficult to annotate NHEJ-mediated variants since NHEJ leaves short random indels rather than specific signatures (14,58,59). In addition, its randomness limits the ability to distinguish NHEJ from other variant formation mechanisms that do not require DSBs (60–62). Thus, they were mostly unidentified by GDBr; further, repetitive regions were excluded. Since the same repetitive variant can be generated through different mechanisms, we could not determine their underlying mechanisms. Finally, some indels (∼11%) and complex substitutions (∼89%) did not show DNA signatures that could be interpreted as HR-mediated repairs. It is important to note that polymerase errors, replication slippage, retrotransposition and other DSB repair mechanisms exhibit distinct repair signatures compared to TMEJ and SSA (Supplementary Figure S9). However, these mechanisms may produce convergent genomic signatures, making it challenging to distinguish them based solely on genomic sequences. Further biochemical analysis of DNA damage and repair mechanisms will help understand and assign these genetic variants and underlying repair mechanisms.

Recent advances in long-read sequencing technologies have helped to accumulate high-quality genomes of many individuals in any species, even for cancer genomes. Specifically, to annotate cancer repair mechanisms, it is necessary to obtain high-quality genome assemblies of both tumour and matched-normal samples, as GDBr uses genome assemblies as inputs. This application of GDBr could help answer important questions about cancer genome evolution, such as how specific HR-mediated mechanisms are involved in cancer DSB repairs, how these mechanisms contribute to structural variation in cancer and what role they play in DSB repair following cancer treatments. Moreover, these HR-mediated DSB repair mechanisms were not enriched along chromosomes in the draft human pangenome; however, their distributions in cancer genomes may differ. GDBr could help identify fragile regions prone to DSBs and annotate their repairs using HR-mediated mechanisms. Unfortunately, we are currently unable to annotate cancer mutations using GDBr due to the lack of public data. This limitation could be addressed as more cancer genome assemblies become available in the near future.

Annotating the underlying DSB repair mechanisms of genetic variants of high-quality genomes with GDBr can help understand how the genome has been shaped by DNA damage and HR mechanisms. Thus, GDBr has the potential to expand our knowledge of mutation accumulation and genome evolution.

## Supplementary Material

gkae1295_Supplemental_Files

## Data Availability

GDBr code used in this analysis is available on Zenodo at https://doi.org/10.5281/zenodo.14063503 (GDBr version 1.0.0), and developed on GitHub (https://github.com/Chemical118/GDBr). GDBr benchmark results using draft pangenome assemblies of HPRC are also available on Figshare at https://doi.org/10.6084/m9.figshare.27644268.v2. The 94 pangenome assemblies that we used in this study are available at https://zenodo.org/record/5826274/files/HPRC-yr1.agc?download=1 (HPRC-yr1.agc for HPRC Year 1 genome assemblies).
