# Supplementary material for: GDBr: genomic signature interpretation tool for DNA double-strand break repair mechanisms
Source: Nucleic Acids Res. 2025 Jan 11;53(2):gkae1295. doi: 10.1093/nar/gkae1295 (PMC11724358; doi:10.1093/nar/gkae1295)
Supplement: gkae1295_Supplemental_Files [file gkae1295_supplemental_files.zip › GDBr_Supplementary_Figures_CleanVersion.docx]

GDBr: Genomic signature interpretation tool for DNA double-strand break repair mechanisms

Supplementary figures

**
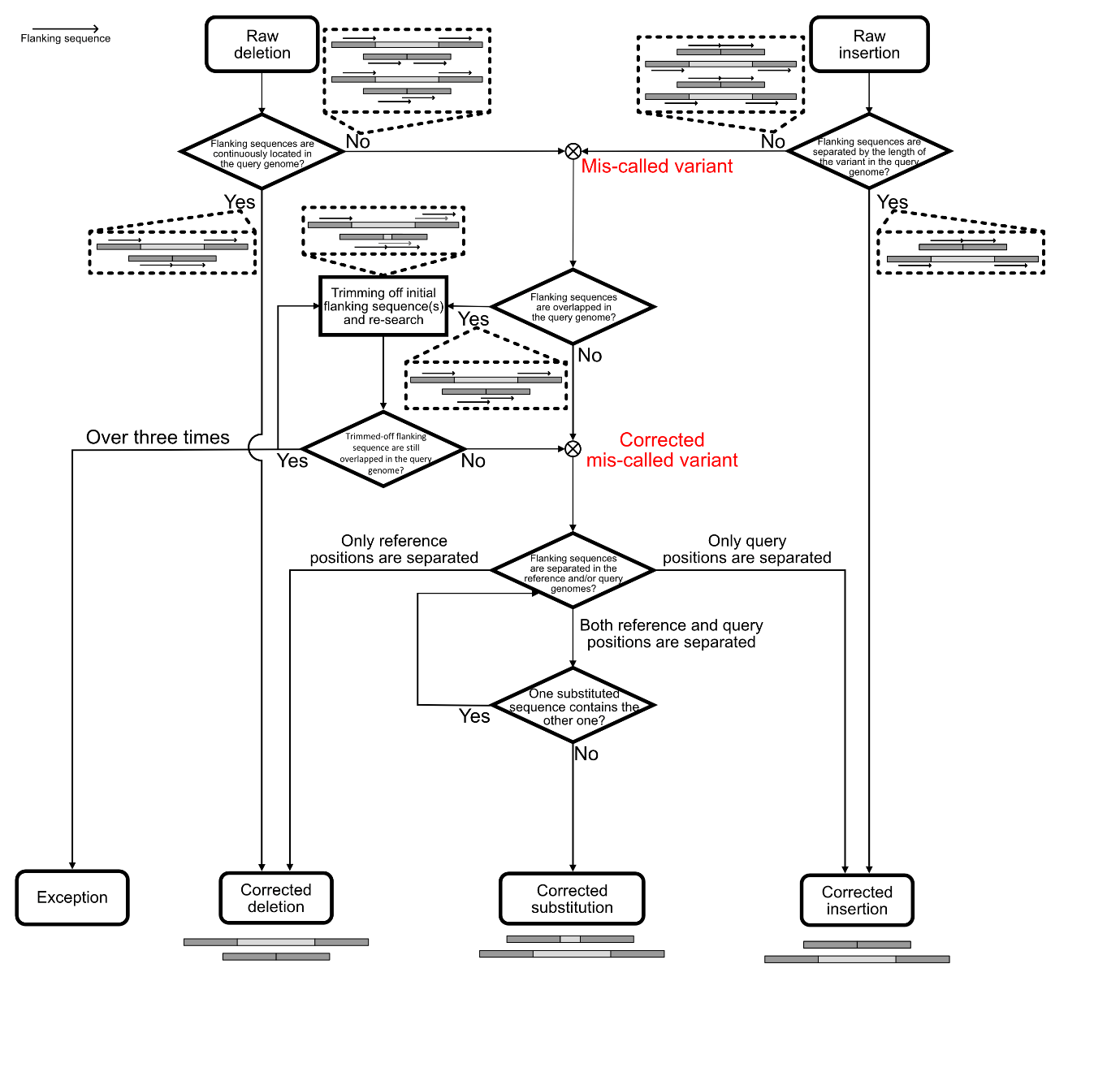
**

**Supplementary Figure S1. Flowchart of variant correction and filtering steps in GDBr.**

**
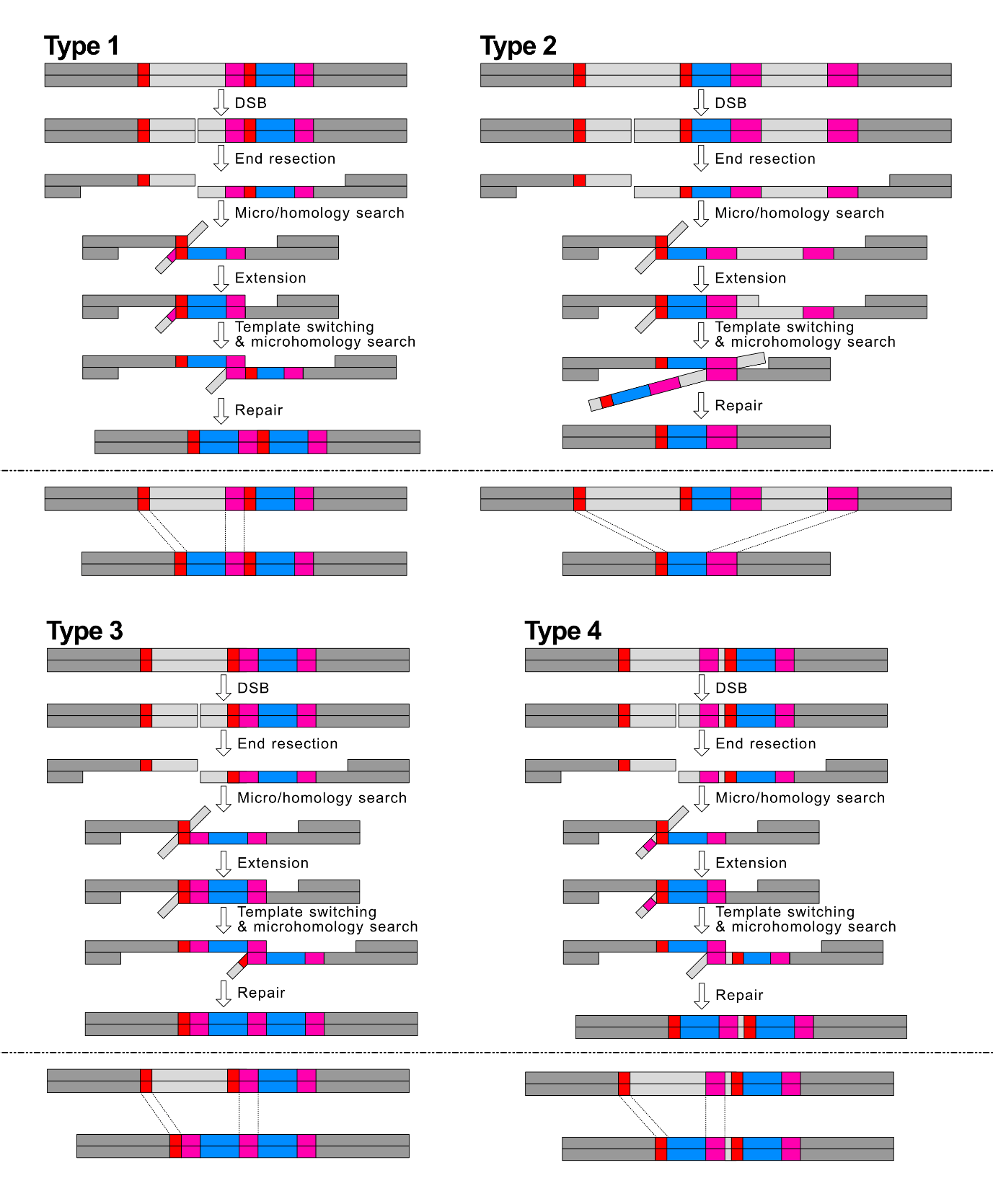
**

**Supplementary Figure S2. Schematic representation of templated insertion types.** These four types were categorized based on the positional combination of the first and second microhomology sequences. Dark grey boxes represent sequences near the variant that remained unchanged during repair. Red and magenta boxes indicate the first and second microhomology sequences, respectively, while light grey boxes represent sequences that were deleted during end resection.

**
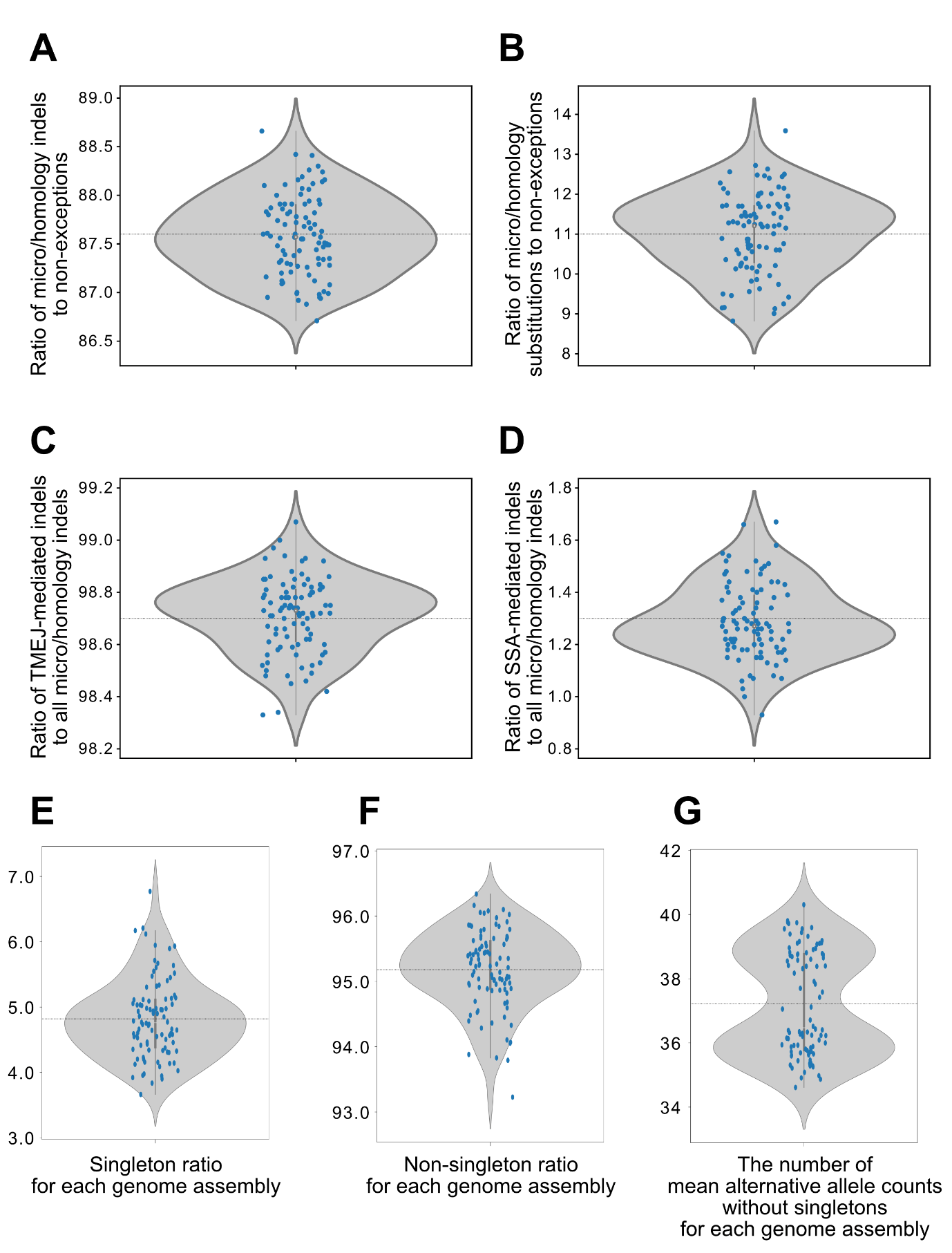
Supplementary Figure S3. Variant classification in 94 draft human pangenome assemblies based on micro/homology in flanking sequences.** (A) Ratio distribution of micro/homology indels to all corrected indels. (B) Ratio distribution of micro/homology substitutions to all corrected substitutions. (C) Ratio distribution of TMEJ-mediated indels to all micro/homology indels. (D) Ratio distribution of SSA-mediated indels to all micro/homology indels. Each dot represents a genome assembly. The horizontal dotted line indicates the ratio calculated by merging the results from the 94 genome assemblies. (E–G) Singleton and non-singleton data across the human draft pangenome.

**
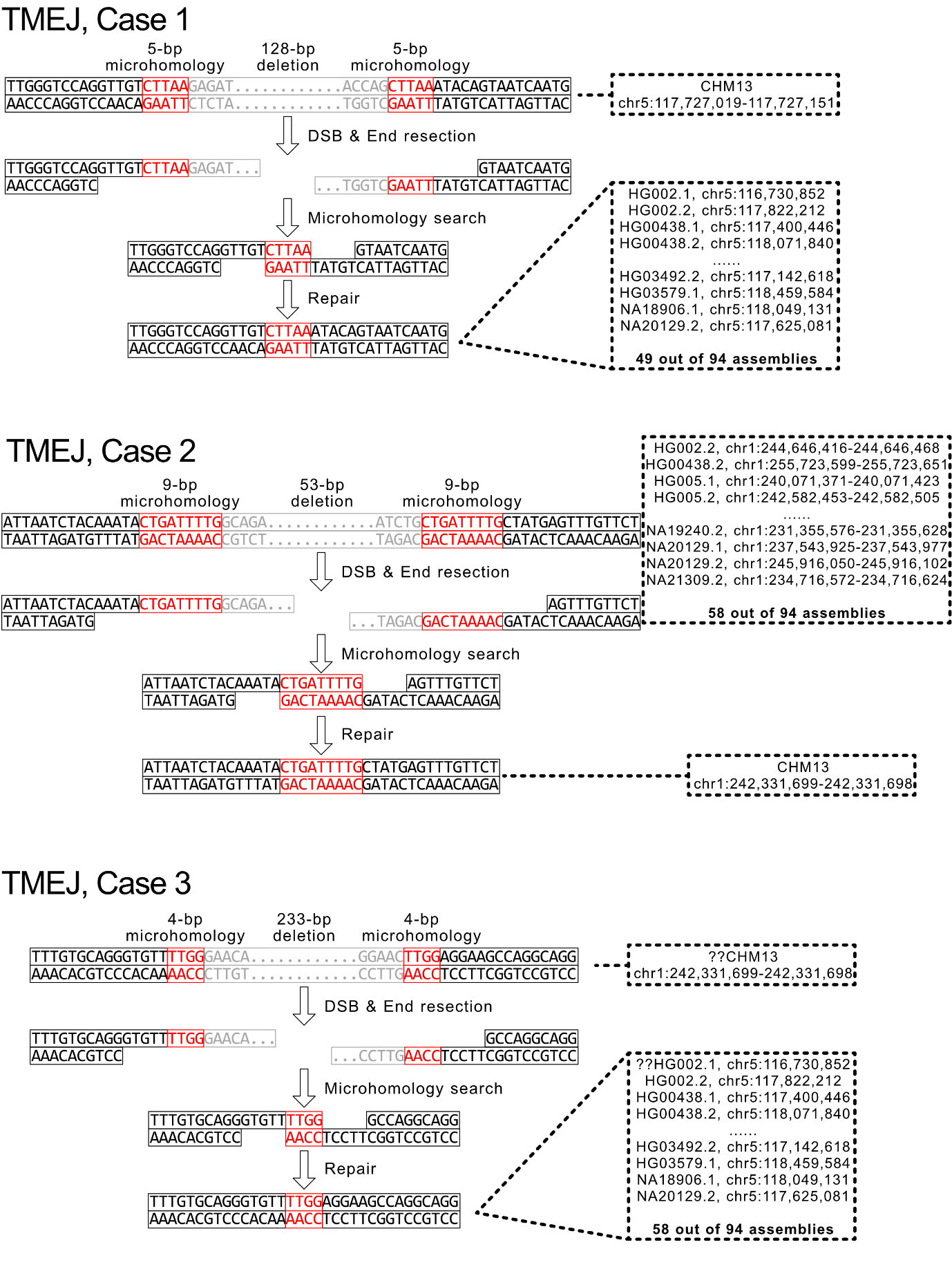
**

**
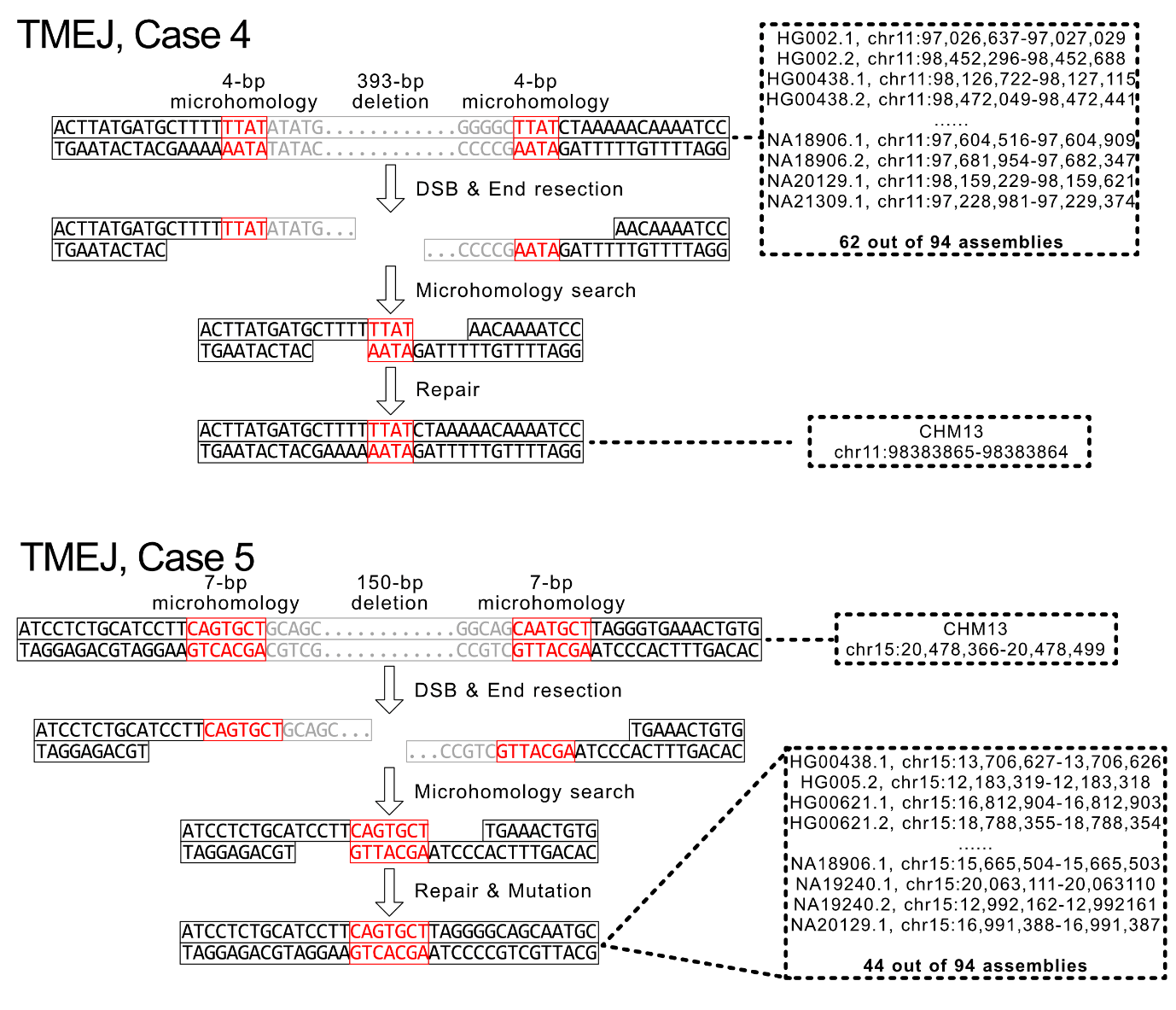
**

**
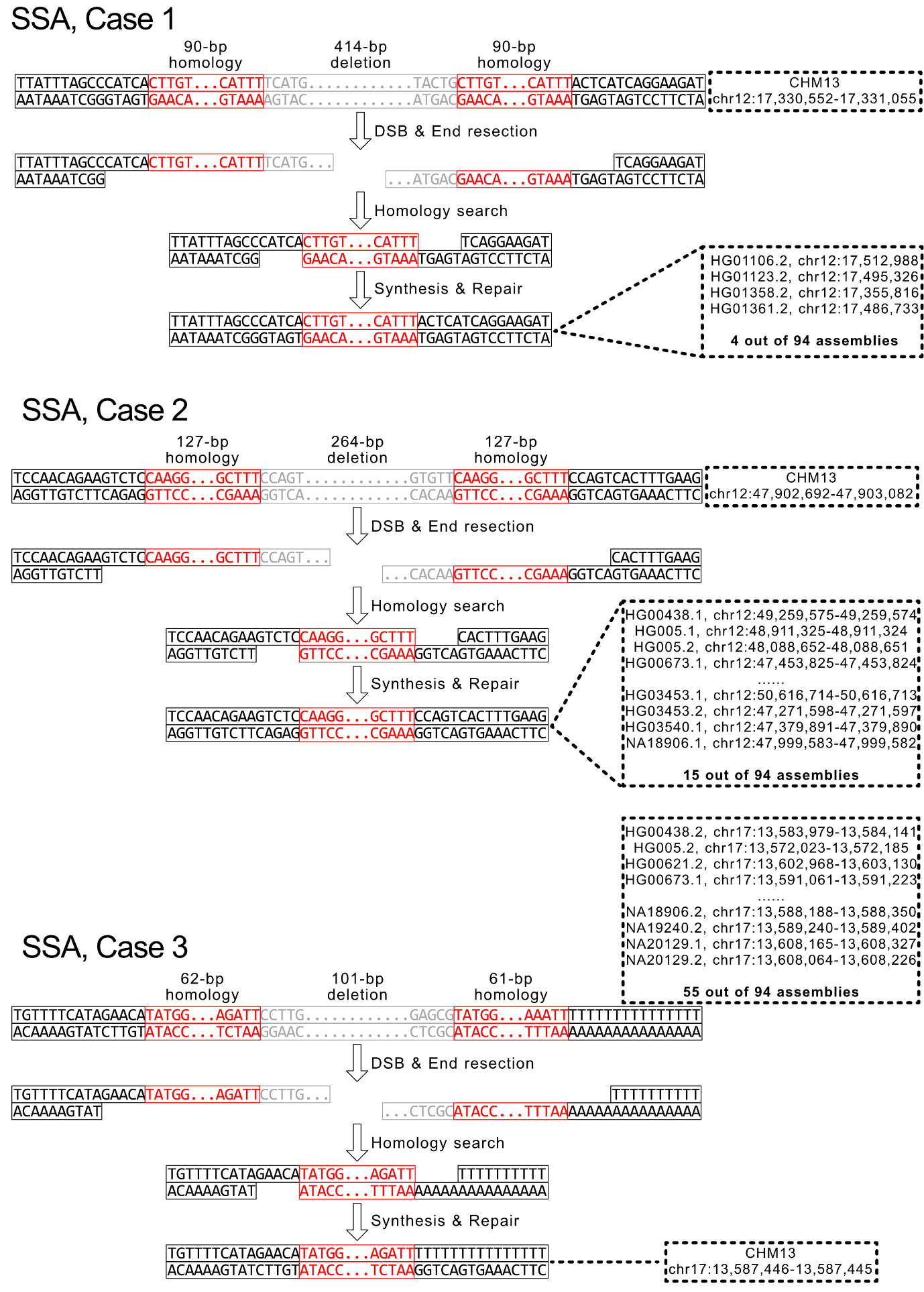
**

**
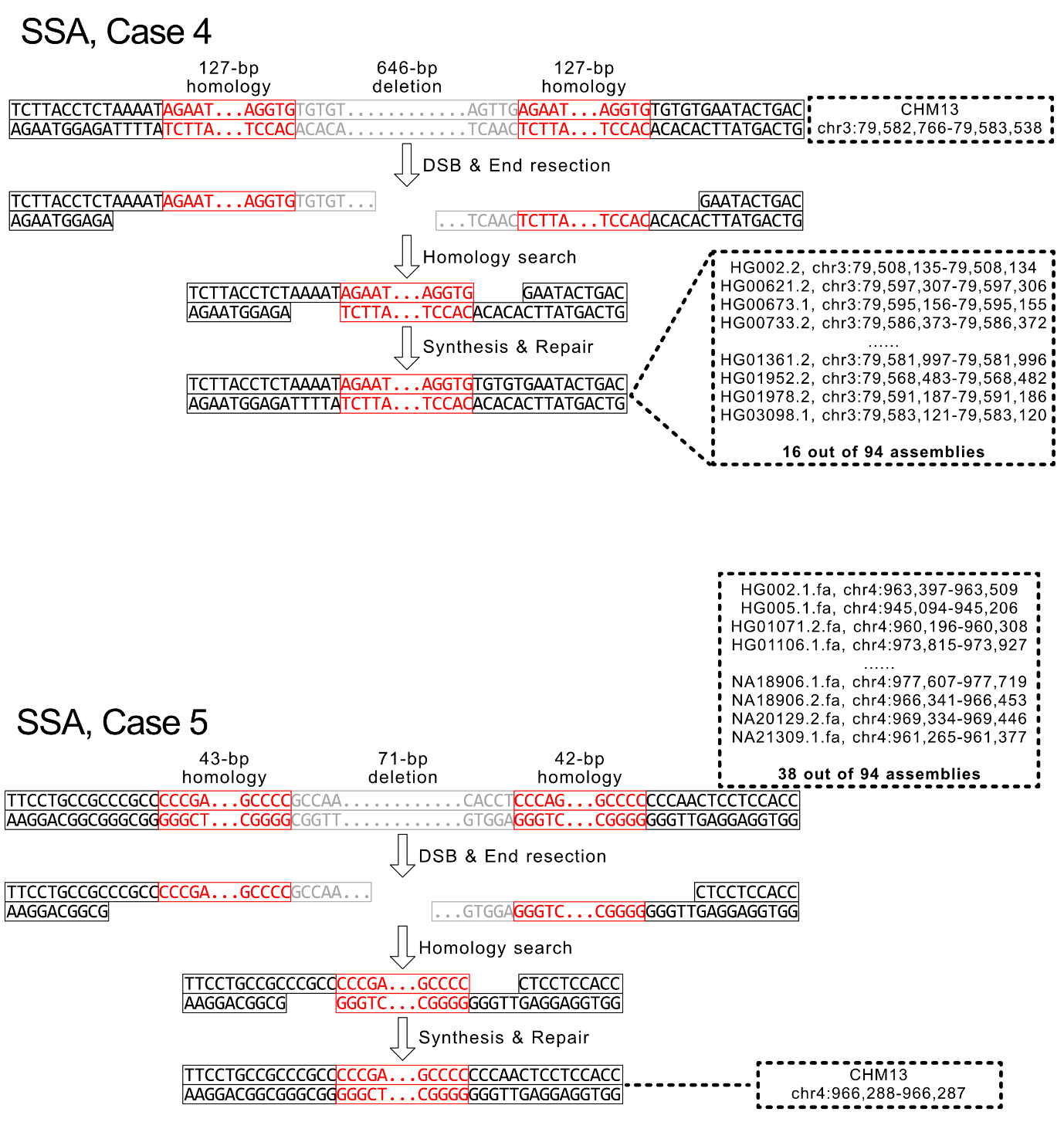
**

**
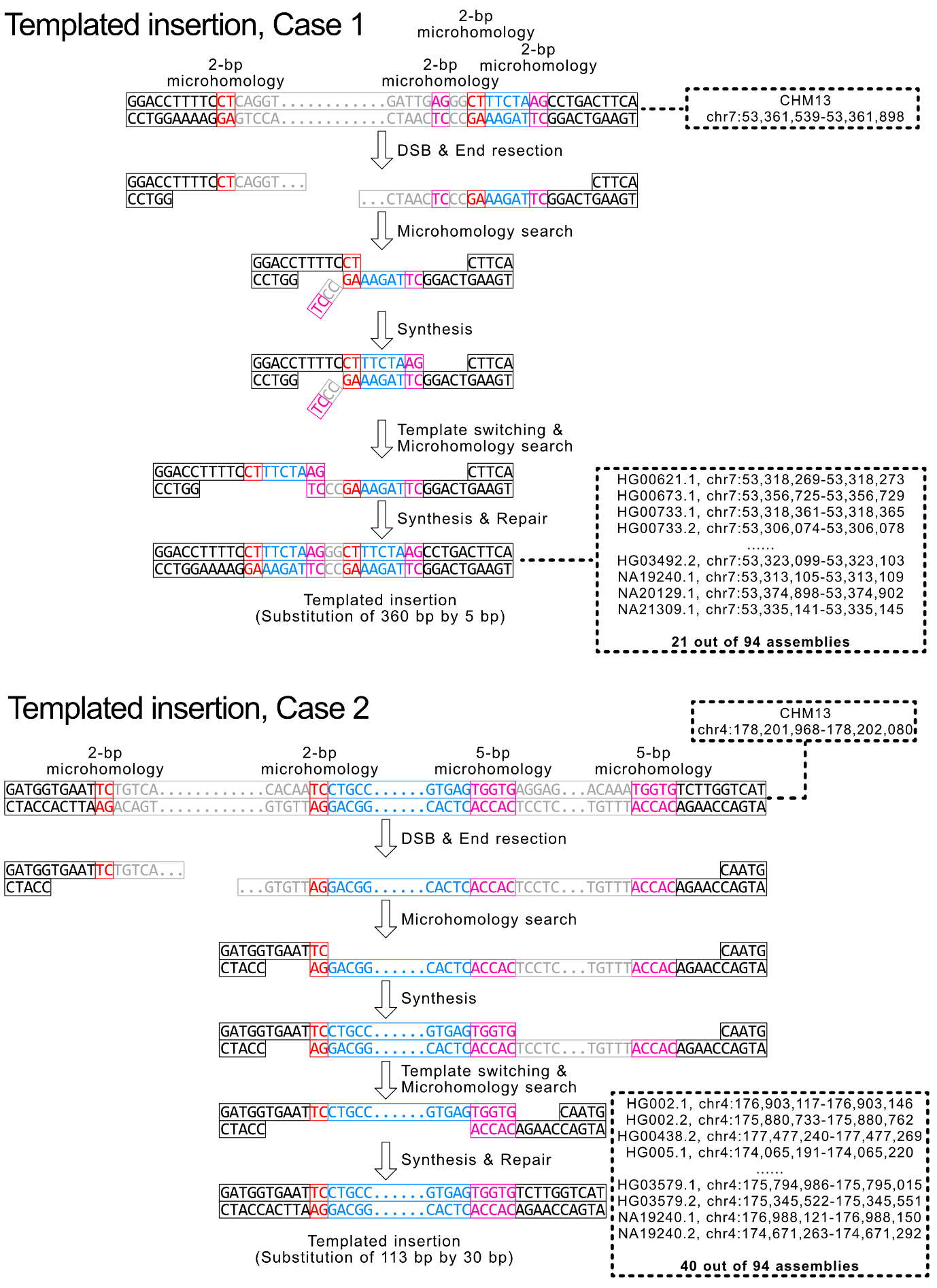
**

**
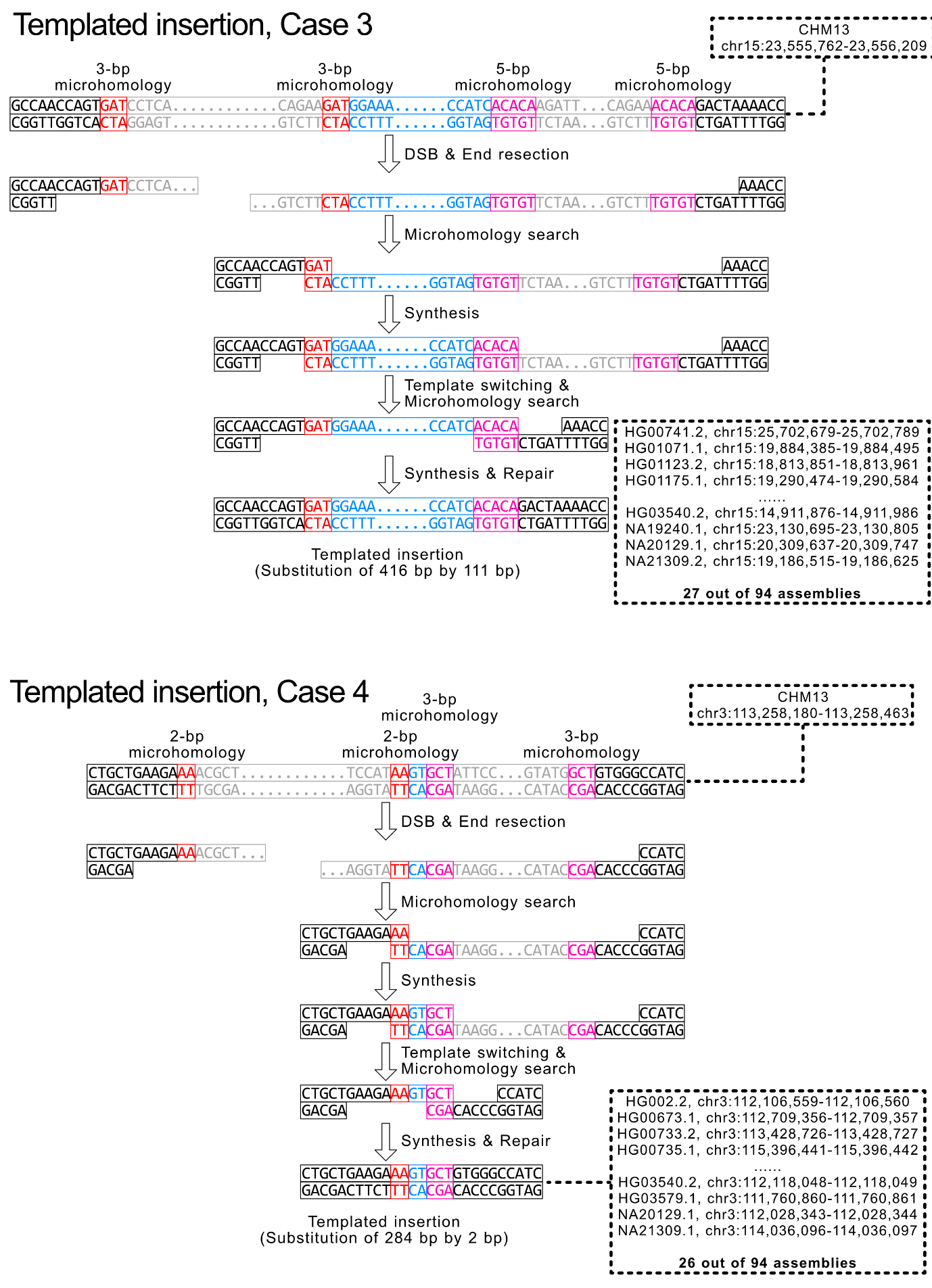
**

**
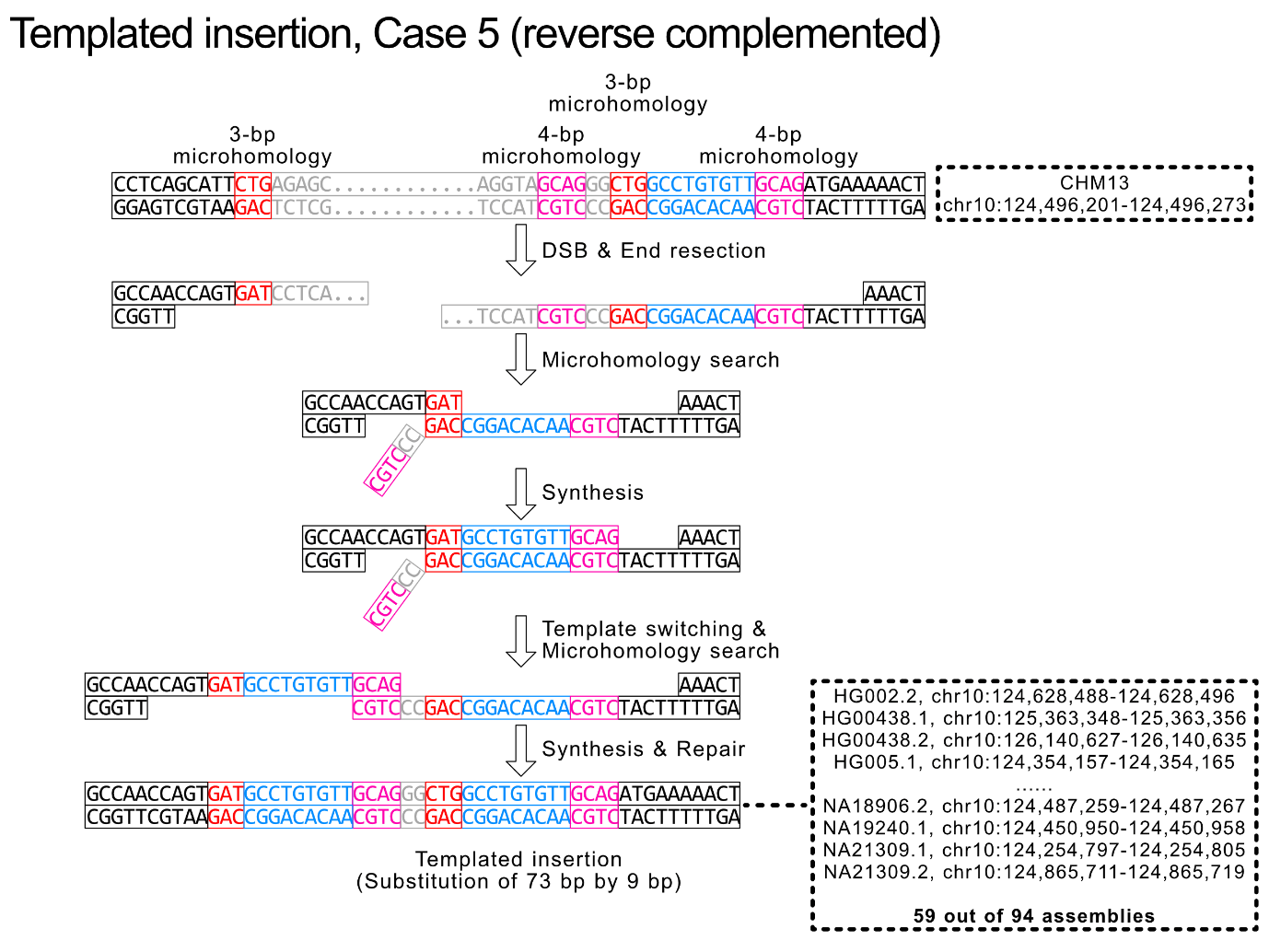
**

**Supplementary Figure S4. Real case examples of indels and complex substitutions identified by GDBr.** The underlying DSB repair mechanisms of these variants were determined based on micro/homology identification and the repair mechanism annotation provided by GDBr. Black boxes represent sequences near the variant not changed during the repair process. Red boxes represent micro/homology sequences. Magenta boxes indicate the second microhomology sequences for templated insertions. Grey boxes represent deleted sequences during end resection. Dotted boxes show variant positions in the reference T2T-CHM13 and query genomes.

**
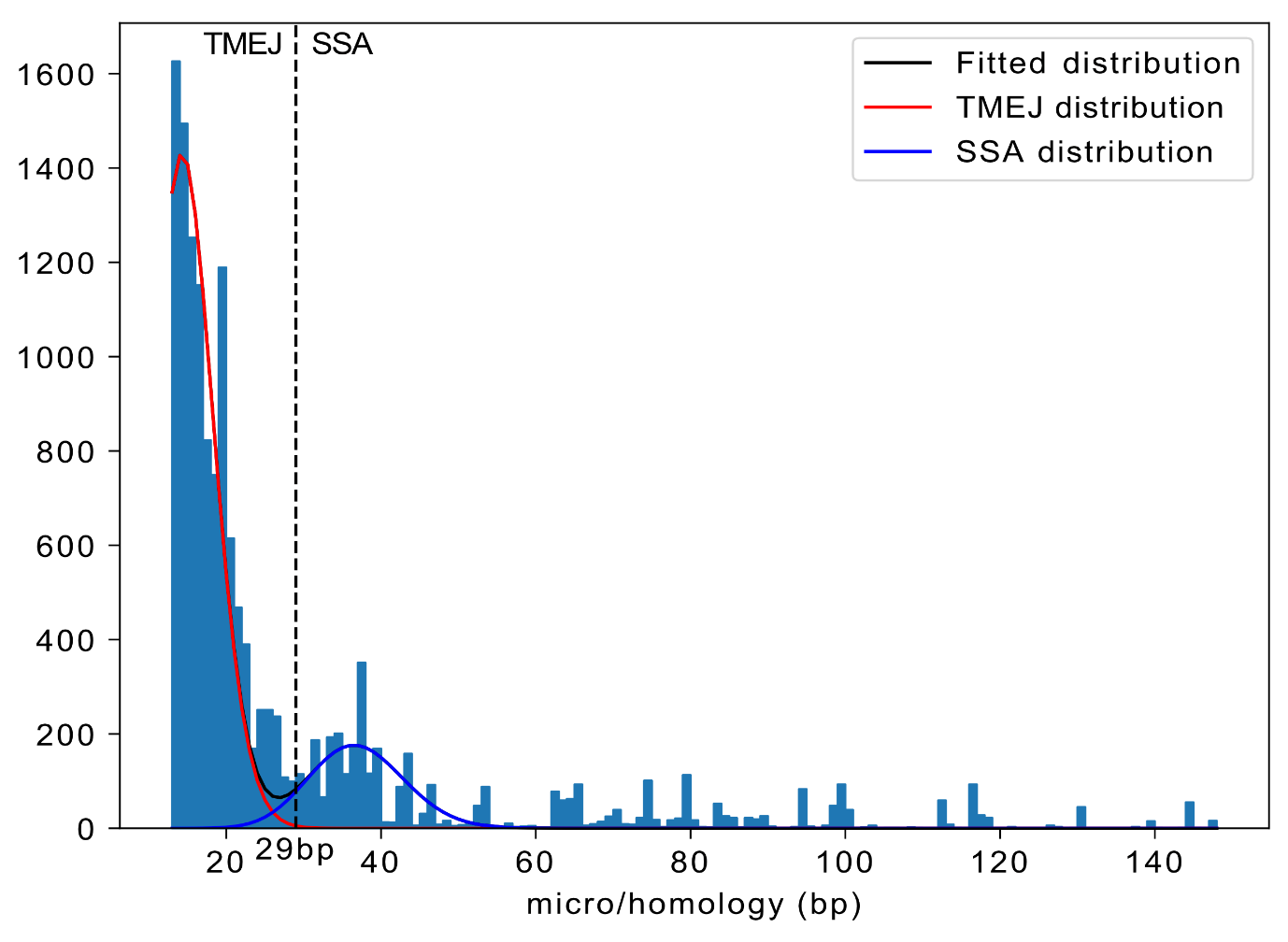
**

**Supplementary Figure S5. Separation of potential micro/homology distributions using two independent Poisson distributions.** The red and blue solid lines represent TMEJ- and SSA-mediated distributions, respectively, while the black solid line represents a fitted distribution obtained by merging the two Poisson distributions.

**
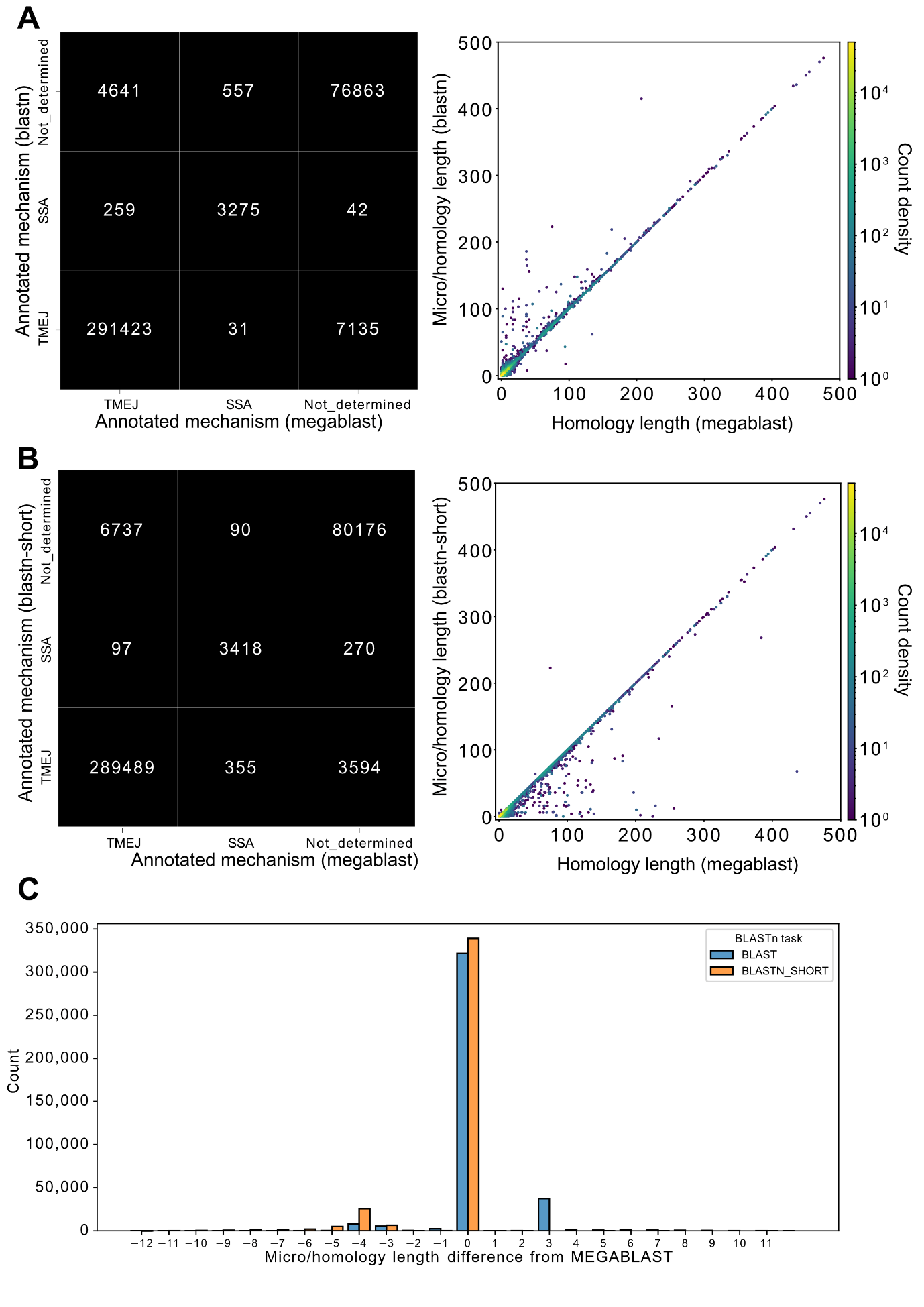
**

**Supplementary Figure S6. Influences of BLASTn parameters.** (A and B) Comparisons of *megablast* vs. *blastn-short* (A) and *megablast* vs. *blastn* (or *dc-megablast*) (B). (C) Differences in micro/homology length identified by *megablast* compared to *blastn* (blue) and *blastn-short* (orange).

**
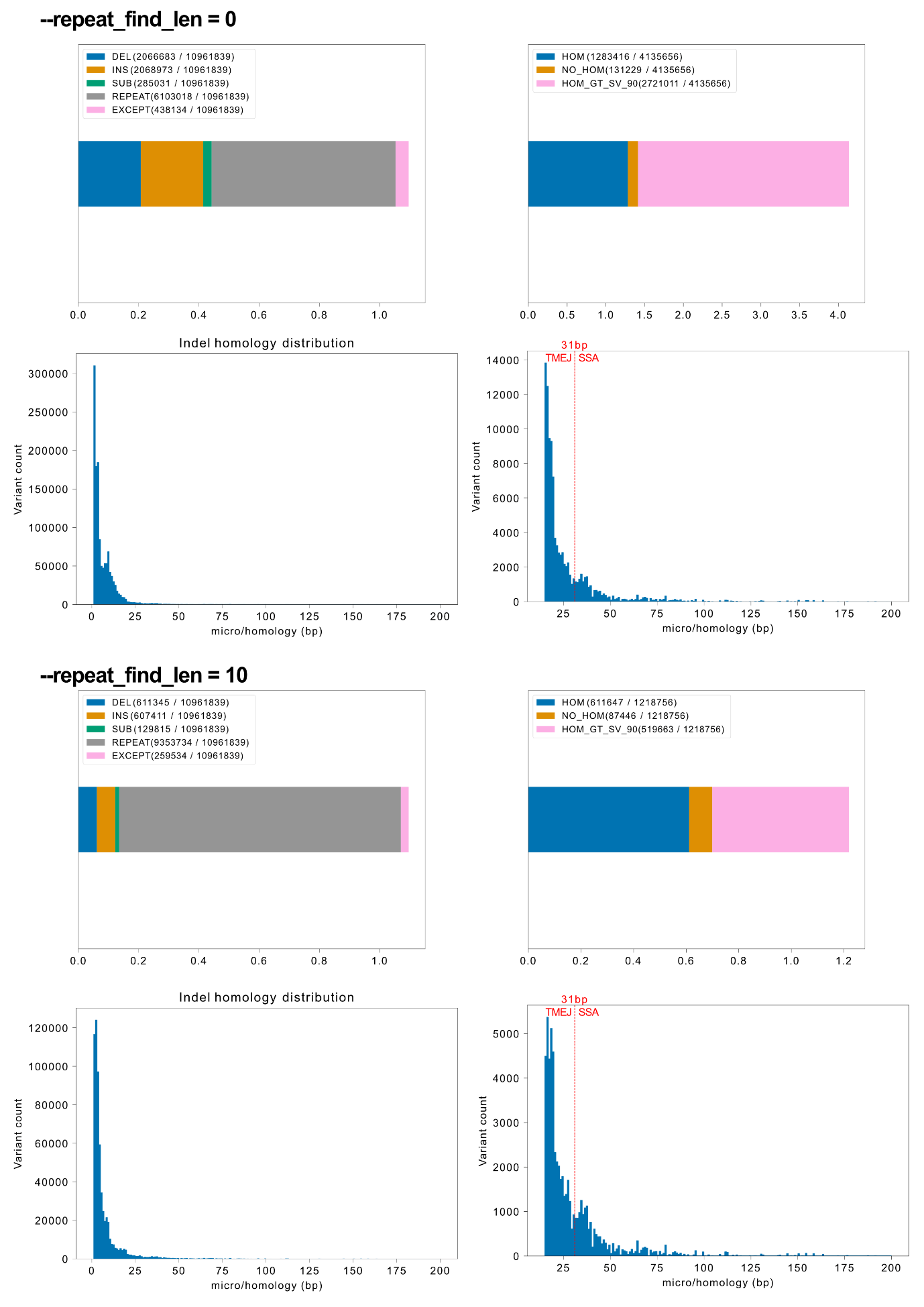
**

**
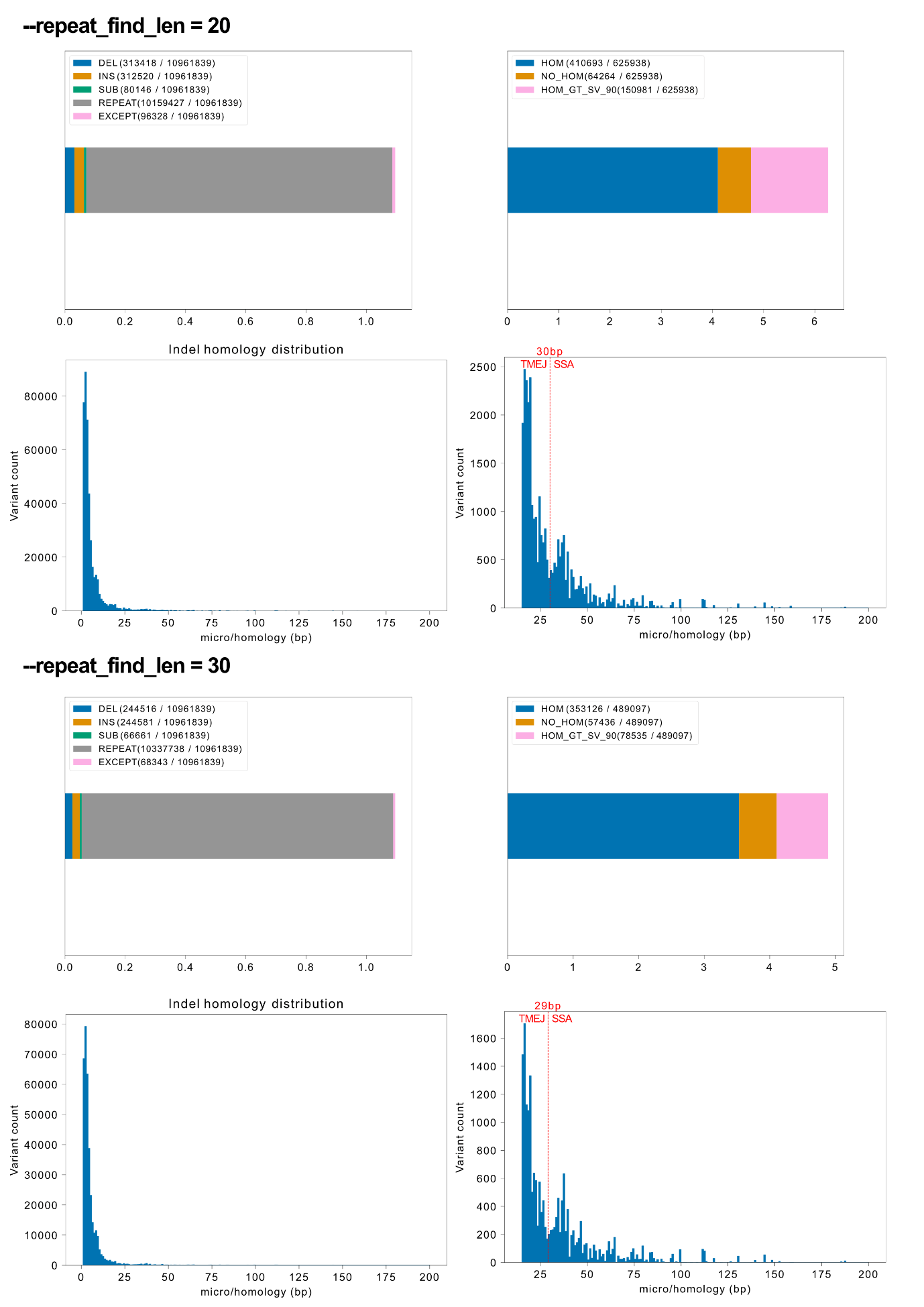
**

**
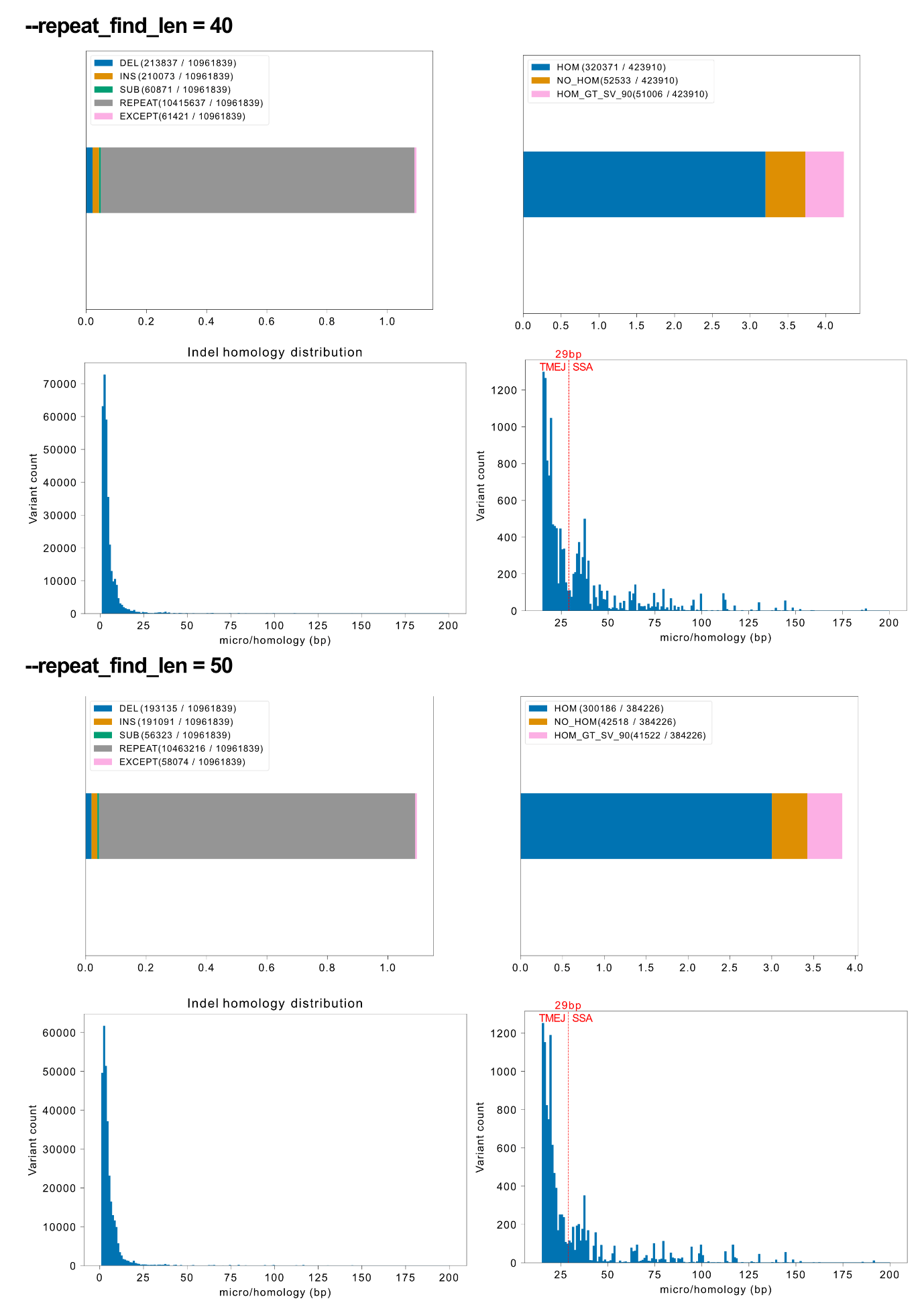
**

**Supplementary Figure S7. Effects of sequence length on repeat search results.** The *--repeat_find_len* parameter was tested at intervals of 10, ranging from 0 to 50 (i.e., 0, 10, 20, 30, 40, 50).


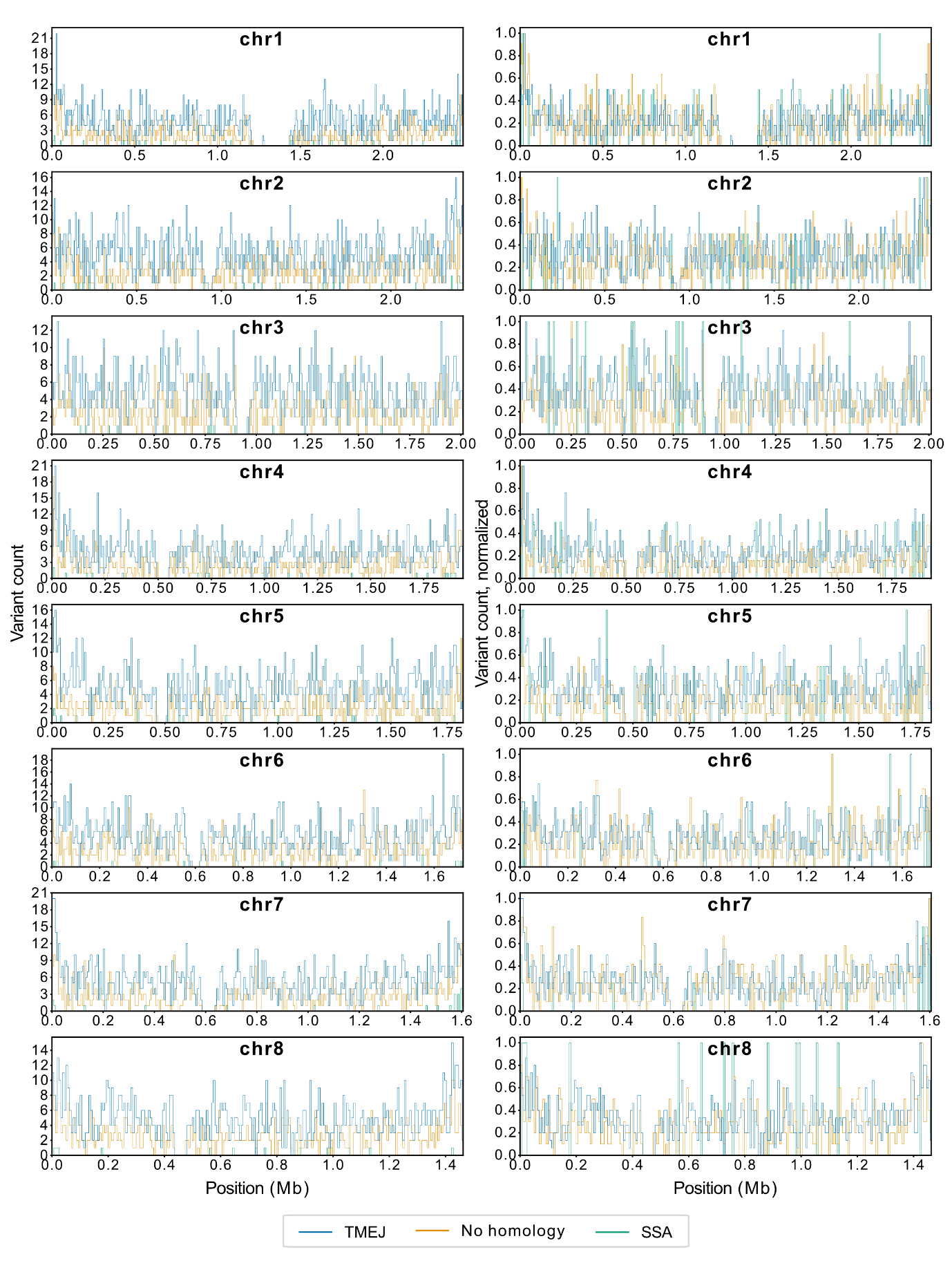


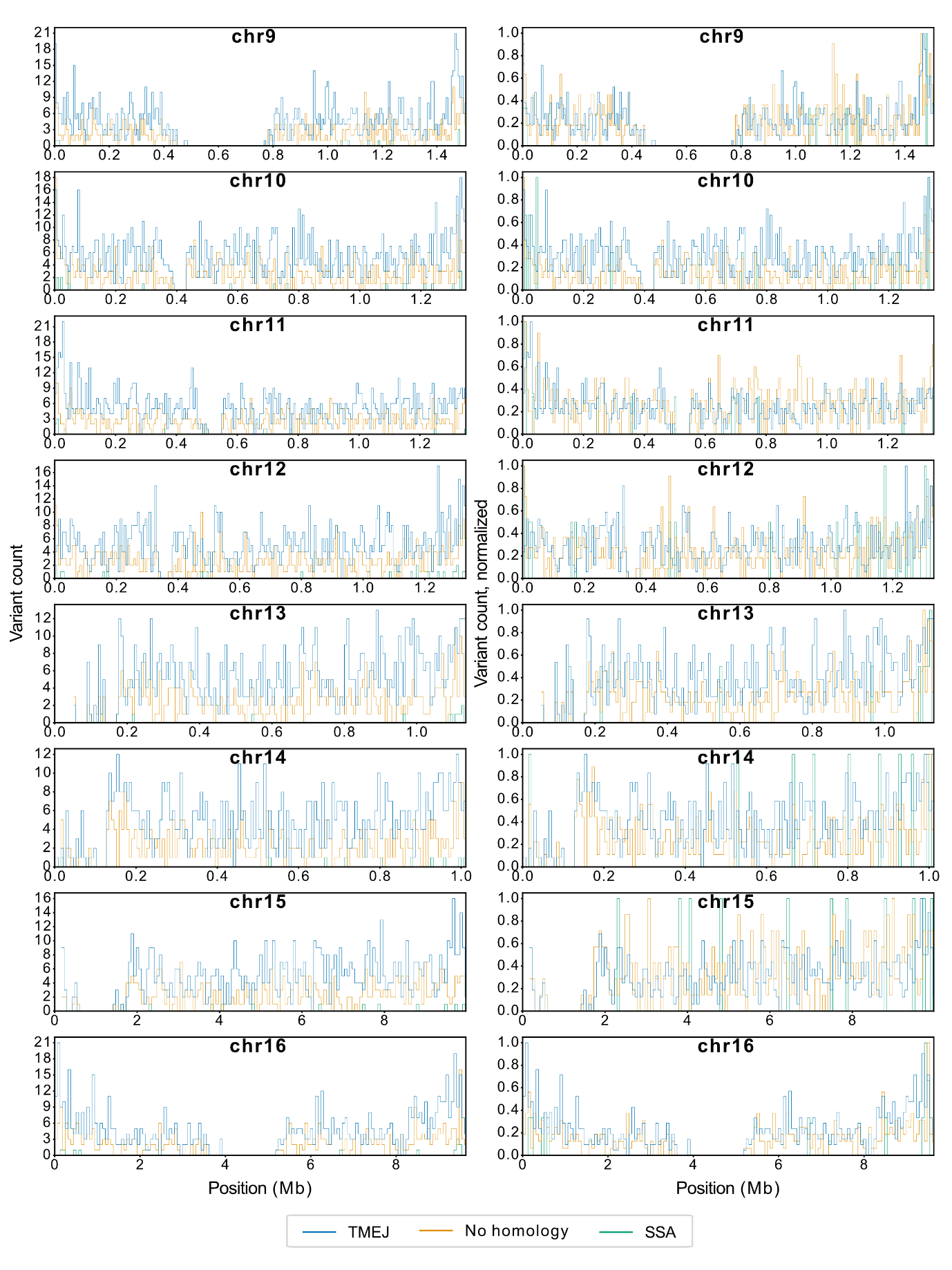


**
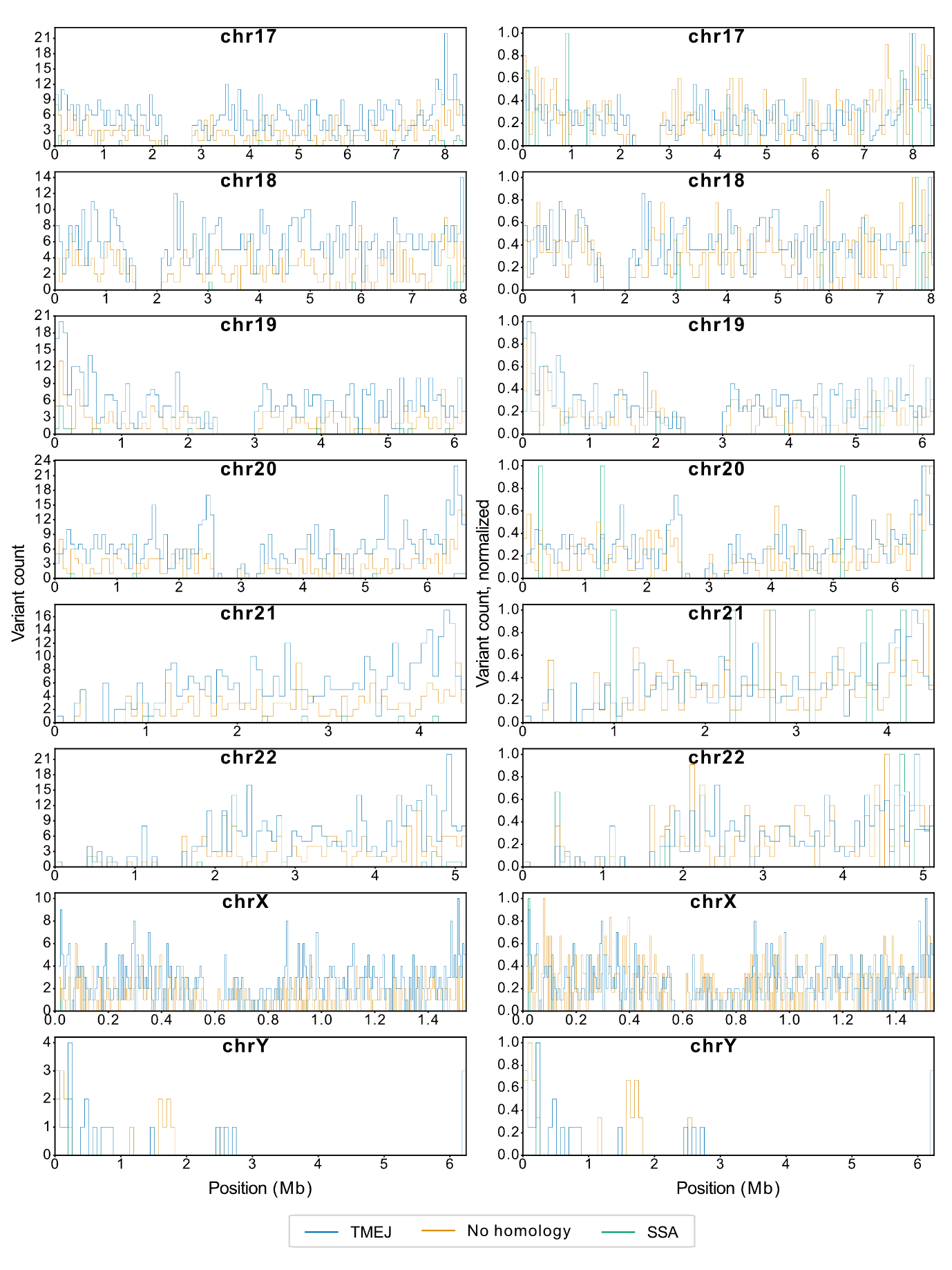
Supplementary Figure S8. Chromosomal distributions of DSB repair events in the human population.** Left panels represent absolute counts of three different DSB repair mechanisms along chromosomes and the right panels show their normalised counts based on the highest counts in each chromosome.

**
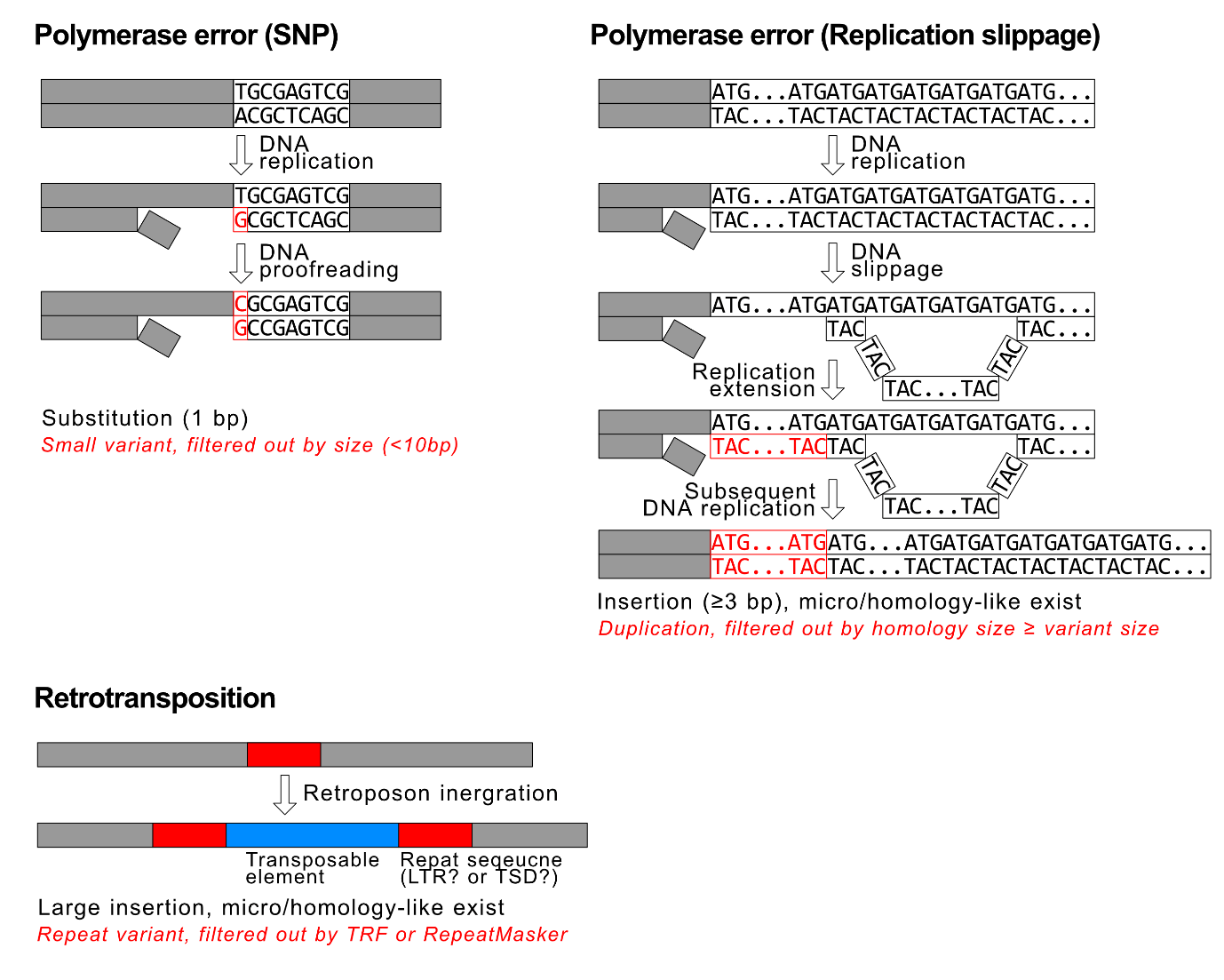
**

**
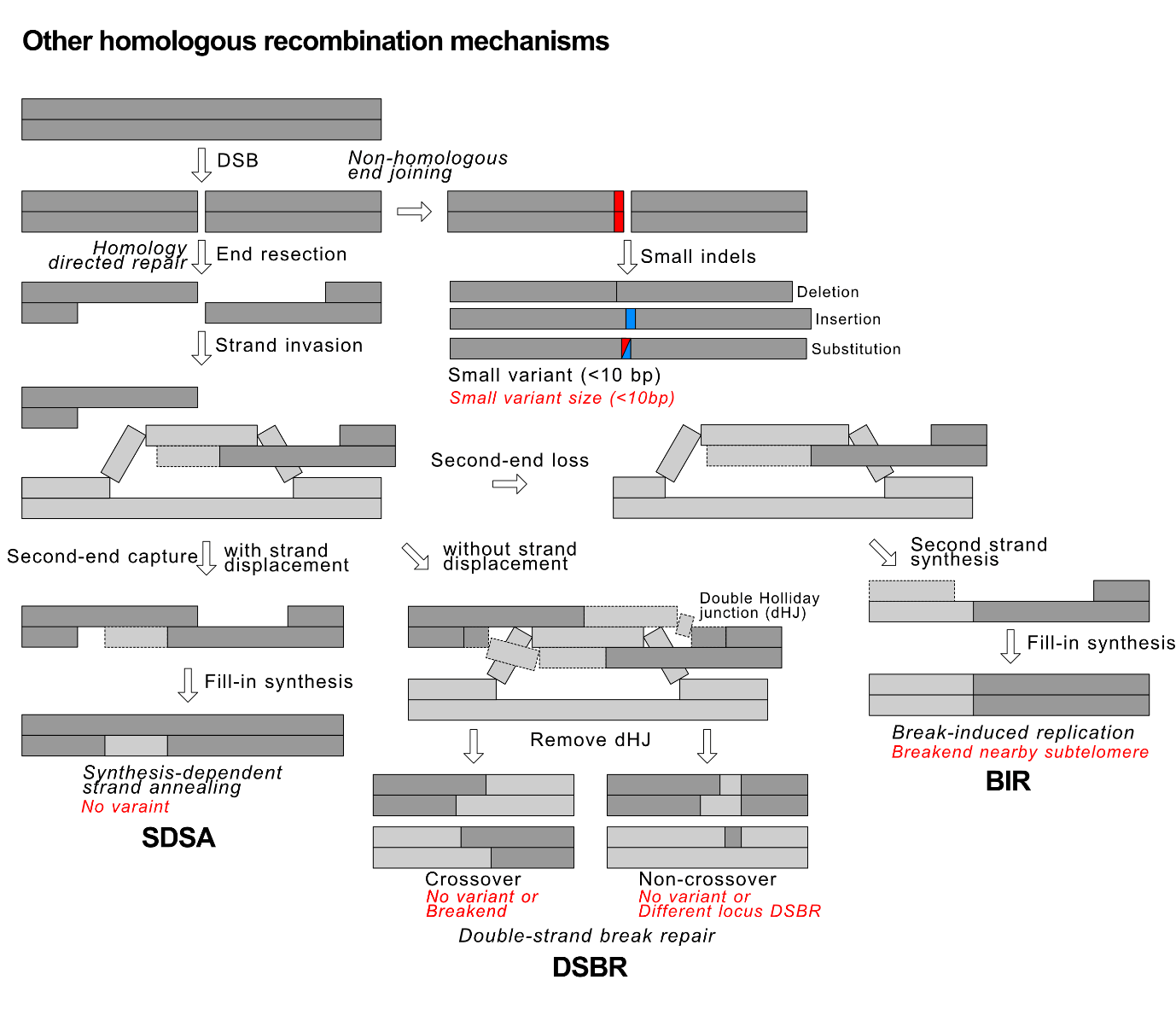
**

**Supplementary Figure S9. Potential repair mechanisms and their distinct genomic signatures.** (A) First, variants can result from DNA polymerase errors. However, these errors typically generate single-nucleotide variants rather than ≥10 bp variants. In addition, while replication slippage of DNA polymerase may cause larger variants by repetitively replicating the same locus, this generally results in repetitive variants rather than non-repetitive ones. Thus, our ≥10 bp, non-repetitive criteria effectively rule out DNA polymerase errors as a source. (B) Second, retrotranspositions can generate variants. However, these typically result in repetitive variants, as retrotransposition and other transposable element activity follow a copy-and-paste mechanism. Again, our criteria exclude this possibility as well.

It is important to note that the underlying repair mechanisms behind repetitive variants are difficult to determine because the same repetitive variant can be produced through various processes. We mentioned it in our manuscript, and this is why we exclude repetitive variants from our analysis. (C) Third, non-homologous end-joining (NHEJ) is another possible mechanism, but it generally produces small (≤5 bp) random indels. Our length criteria exclude these small indels. Larger variants are typically generated by homology-directed repair (HDR) mechanisms after double-strand breaks (DSBs), which involve resecting the damaged ends before repair.

It is also worth noting that we did not filter variants based on micro/homology presence or absence. Our filtering was solely based on length (≥10 bp) and non-repetitive criteria. Nevertheless, the majority of our filtered variants exhibit micro/homology signatures (78.1% with micro/homology, 11.1% without micro/homology, and 10.8% as exceptions), further supporting the idea that these larger variants arise through HDR mechanisms. (D) Finally, while other HDR mechanisms could provide alternative explanations, such as synthesis-dependent strand annealing (SDSA), double-strand break repair (DSBR), and break-induced replication (BIR), their signatures exclusively differ from those of TMEJ and SSA. SDSA and DSBR, for instance, replace original sequences into non-allelic sequences, whereas TMEJ and SSA are characterized by sequence deletions. Therefore, these mechanisms would not be mistaken for one another. We could further annotate these replacement events using GDBr by turning on --diff_locus_dsbr_analysis. BIR is also not confused with TMEJ and SSA because it requires single-ended DNA damage, which generates variants only at the ends of chromosomes. The variants we identified are located in the internal chromosomal regions, rather than the telomeric or subtelomeric regions.

In summary, while GDBr might fail to identify the exact underlying mechanisms of some ≥10 bp, non-repetitive variants, the TMEJ and SSA annotations are unlikely to be confused with other mutagenic mechanisms.
